# The growth, lipid accumulation and adaptation mechanism in response to variation of temperature and nitrogen supply in psychrotrophic filamentous microalga *Xanthonema hormidioides* (Xanthophyceae)

**DOI:** 10.1186/s13068-022-02249-0

**Published:** 2023-01-19

**Authors:** Baoyan Gao, Jian Hong, Jiamin Chen, Hu Zhang, Ren Hu, Chengwu Zhang

**Affiliations:** Department of Ecology, Research Center for Hydrobiology, Jinan University, Guangzhou, 510632 People’s Republic of China

**Keywords:** Psychrotrophic microalgae, *Xanthonema hormidioides*, Lipids accumulation, Adaptation mechanism, Proteomics analysis

## Abstract

**Background:**

Microalgae are promising feedstocks for production of renewable biofuels and value-added bioproducts. Temperature and nitrogen supply are important environmental and nutritional factors affecting the growth and metabolism of microalgae, respectively. In this study, the growth and lipid accumulation of filamentous microalgae *Xanthonema hormidioides* under different temperatures (5, 7, 10, 15, 20, 25, 27 and 30 °C) and initial nitrogen concentrations (3, 9, 18 mM) were investigated, and its adaptive mechanisms of tolerance to low temperature and nitrogen stress were analysis by proteomics.

**Results:**

The optimum temperature range for the growth of *X. hormidioides* was between 15 and 20 °C, and the algal cells had slow growth rate at 5 °C and could not survive at 30 °C. The maximum biomass concentration was 11.73 g L^−1^ under the temperature of 20 °C, and the highest total lipid content was 56.63% of dry weight. Low temperature did not change the fatty acids profiles but promoted the accumulation of unsaturated fatty acids of *X. hormidioides*. The maximum contents of palmitoleic acid, eicosapentaenoic acid and total fatty acid were 23.64%, 2.49% and 41.14% of dry weight, respectively. Proteomics was performed under three temperature (7, 15, 25 °C), two nitrogen concentrations (3 and 18 mM) and two cultivation times (day 3 and 12). A total of 6503 proteins were identified. In the low temperature, photosynthesis-related proteins were down-regulated to protect the photosynthetic apparatus. The up-regulation of key enzymes DGAT and PDAT demonstrated the accumulation of TAGs under low nitrogen treatment. The proteins related to ribosome, phosphatidylinositol signaling system, antioxidant system and cold shock proteins (CSPs) in *X. hormidioides* were co-upregulated under the treatment of low temperature, which can alleviate the damages induced by temperature stress and maintain the normal growth and metabolism of algal cells.

**Conclusions:**

*X. hormidioides* is a psychrotolerant microalga. It is an oleaginous filamentous microalga containing hyper palmitoleic acid and a certain amount of eicosapentaenoic acid with great potential for biofuel development, as well as for applications in nutritional health products and other industries.

**Supplementary Information:**

The online version contains supplementary material available at 10.1186/s13068-022-02249-0.

## Introduction

Microalgae are rich in lipids, and diversified high-value intrinsic compounds (e.g., unsaturated fatty acids, and pigments), which are not only high potential resources of bioenergy and biofuels, but also have broad application in human and animal health fields [1]. The need for sustainable energy and healthy bioproducts could drive the future of microalgal biotechnology. Temperature is one of the major environmental factors that affect microalgal growth, biochemical composition and nutrients consumption [2]. The cultivation of microalgae can be carried out over a wide range of temperatures, depending on the species, region, and season [3].

The largest biosphere on Earth is made up of microorganisms that grow in cold environments [4]. In general, the microorganisms isolate from cold habitats which can adapt well to temperatures between 0 and 15 °C. Depending on the degree of adaptation, cold-adapted microorganisms can be classified as psychrophilic and psychrotrophic (psychrotolerant) organisms [5, 6]. Psychrophiles have optimal growth temperatures at or below 15 °C, and maximum growth temperatures below 20 °C. Psychrotrophs/psychrotolerants exhibit the ability to grow at low temperatures, but exhibit optimal growth temperatures above 15 °C [7]. It turns out that these cold-adapted microorganisms are more economical and environment-friendly than those growing at normal or higher temperatures [6]. Meanwhile, these cold strains have been adapted to specific light conditions, and can achieve biomass production at low temperatures [1]. Thus, they can be cultivated outdoors during the cold season or in cultivation facilities in cold regions, which minimize expensive heating and artificial lighting [1]. Mou et al. [8] and Zou et al. [9] also reported that psychrophilic green algae have higher levels of lipids accumulation compared to their mesophilic counterparts, which makes them attractive and promising feedstock for biofuel production. Furthermore, psychrophilic microalgae maintain their membrane fluidity by incorporating higher levels of polyunsaturated fatty acids (PUFA) in membrane lipids at low temperature, making them a potentially useful source of PUFA such as eicosapentaenoic acid (EPA), arachidonic acid (AA) and docosahexaenoic acid (DHA) [10]. In order to improve the economic viability of microalgae-based biodiesel and bioproducts, cold-adapted algae that can rapidly produce large amounts of valuable biocomponents may be an effective approach.

Recently, there has been increased interest on filamentous microalgae due to their strong anti-predation ability, and ease of harvesting in large-scale production [11]. Previous studies of filamentous microalgae *Tribonema* confirmed their potential in wastewater treatment and biofuels production, as well as biorefinery feedstocks with bioproducts [12]. *Xanthonema hormidioides*, like *Tribonema*, is a filamentous yellow-green microalga belonging to the class of Xanthophyceae (Additional file 1: Figure S1). From a pre-study of *X. hormidioides*, we found that it can grow and accumulate high content of lipid and palmitoleic acid at low temperature (< 15 °C) and low nitrogen concentration, so this work aimed to evaluate the growth, lipid and palmitoleic acid production potential of cold-adapted filamentous microalga *X. hormidioides* at different temperatures and nitrogen concentrations. To further understand the metabolic and low temperature adaptation mechanism of *X. hormidioides*, proteomic analysis under different temperatures and nitrogen concentrations were conducted.

## Results

### Effects of different temperatures and nitrogen concentrations on the growth of *Xanthonema hormidioides*

**Fig. 1:**
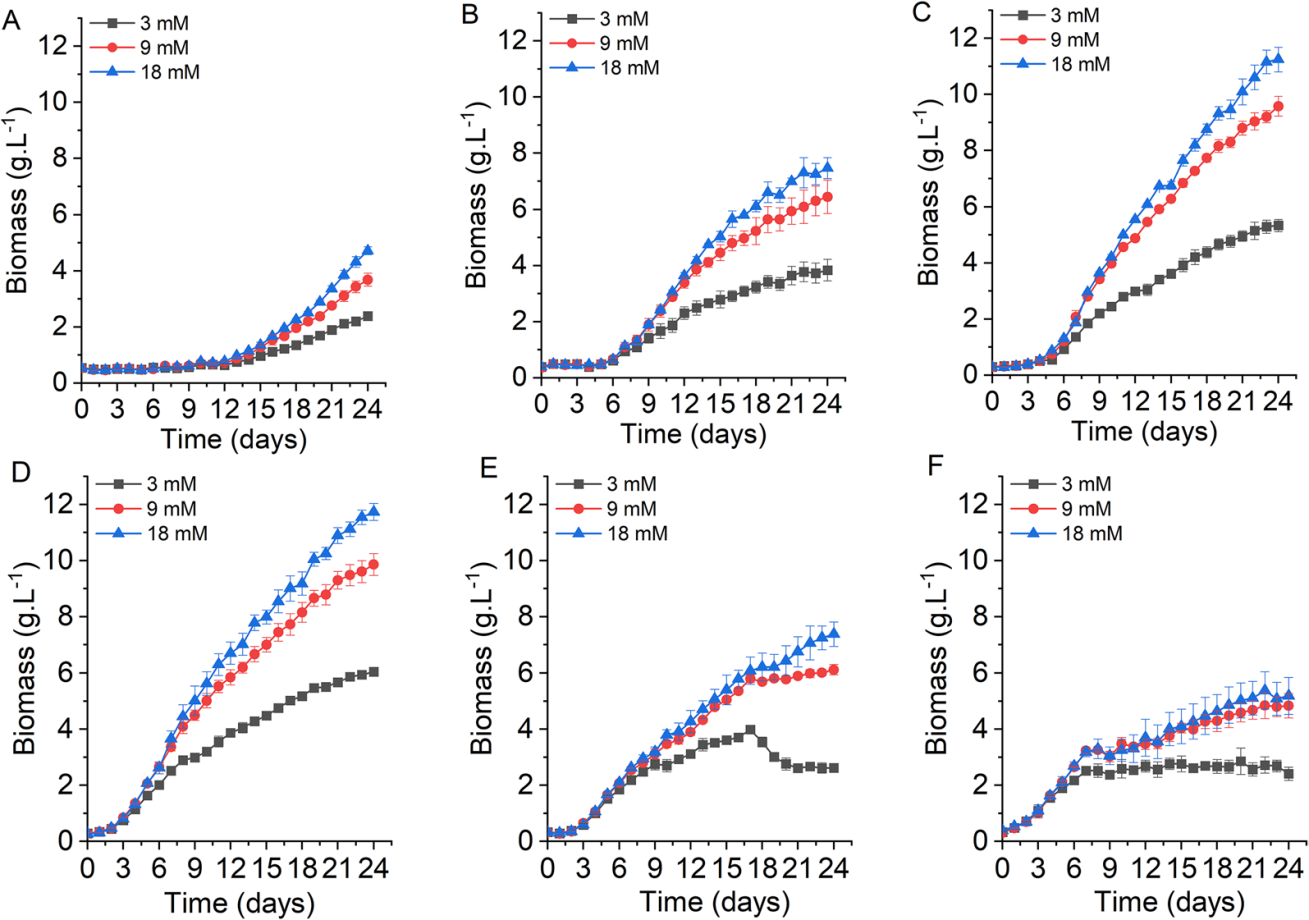
The effect of three nitrogen concentrations on the growth of *X. hormidioides* under different temperatures; **A**, 7 °C; **B**, 10 °C; **C**, 15 °C; **D**, 20 °C; **E**, 25 °C; **F**, 27 °C

The growth of *X. hormidioides* was varied at eight temperatures (5, 7, 10, 15, 20, 25, 27 and 30 °C) and three initial nitrogen concentrations (3, 9 and 18 mM nitrogen treatment) (Fig. 1, Additional file 1: Figure S2). The growth process of *X. hormidioides* required a period of adaptation when the cell was transferred from room temperature to low temperature, and the lag phase was longer as the temperature decreased. The lag phase at 20, 15, 10 and 7 °C, was 3, 5, 7 and 13 days, respectively. At 5 °C, the adaptation time was the longest, and it did not recover until the end of the culture cycle (Additional file 1: Figure S2A). It does not need adaptation time to change from room temperature to 25 °C or high temperature culture. However, at 30 °C, the algal cultures on the 5th day were dead, and the lysis of cell was observed under the microscope (Additional file 1: Figure S2B). Therefore, it could not tolerate high temperatures of 30 °C or above. Under three different nitrogen treatments, the biomass concentration of *X. hormidioides* increased with nitrogen concentration at different temperatures. At the end of culture, the biomass concentration of *X. hormidioides* at 18 mM nitrogen treatment was as follows: 20 °C > 15 °C > 10 °C > 25 °C > 27 °C > 7 °C > 5 °C > 30 °C, and the maximum biomass concentration was 11.73 g L^−1^.

### Effects of different temperatures on consumption rate of total nitrogen and total phosphorus in culture medium

**Fig. 2:**
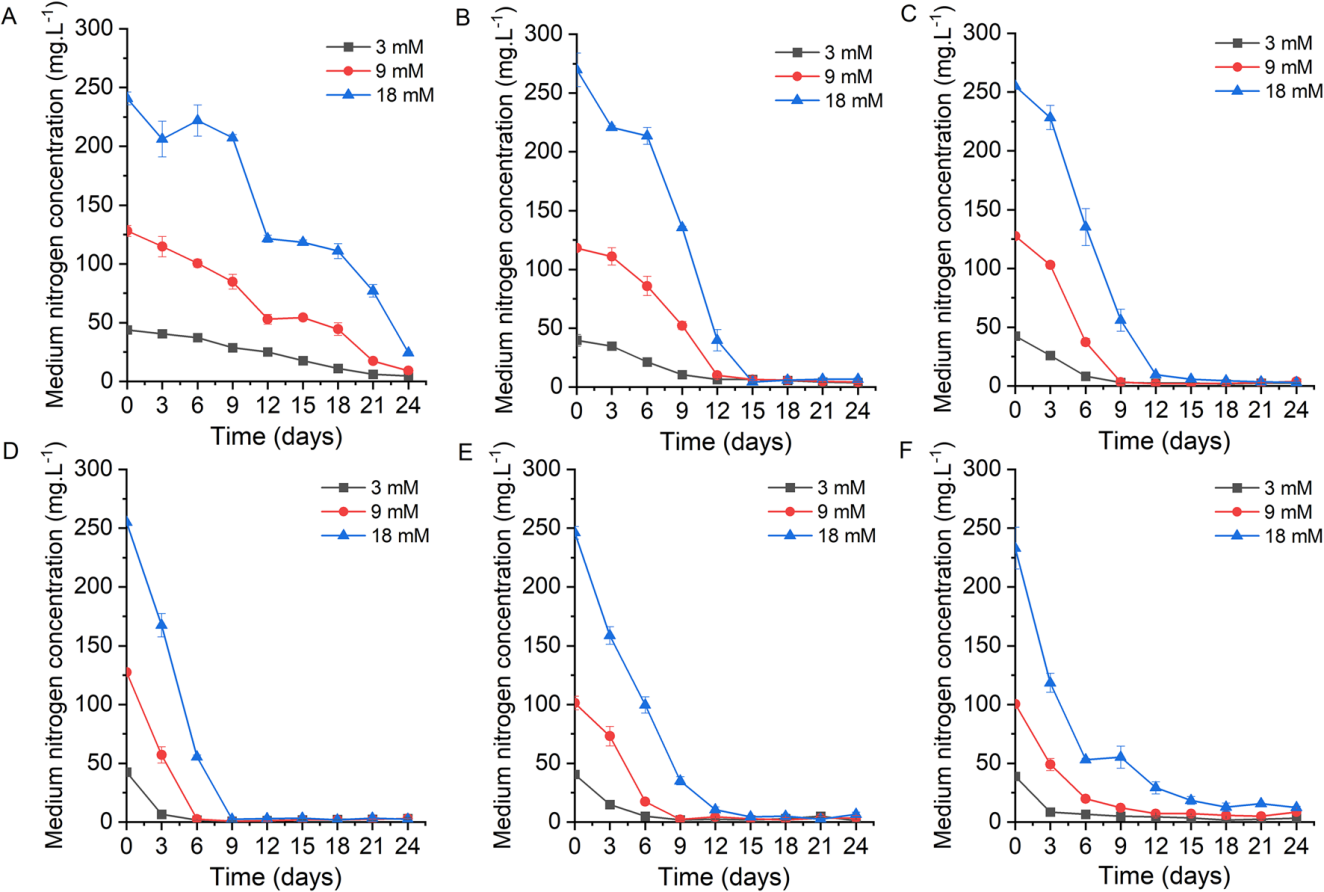
The effect of different temperature on the consumption of medium nitrogen concentration; **A**, 7 °C; **B**, 10 °C; **C**, 15 °C; **D**, 20 °C; **E**, 25 °C; **F**, 27 °C

Under the culture of various temperatures, *X. hormidioides* can uptake urea as nitrogen source in the medium, and the total nitrogen concentration in the medium gradually decreased with time until it was completely absorbed (Fig. 2). Under the condition of 5 °C, the uptake rate of total nitrogen in the medium was slow, and there were about 60% of the original nitrogen concentration remaining in the medium at the end of the culture (Additional file 1: Figure S2C). At 7 °C, the total nitrogen concentration in the medium decreased with time, and the nitrogen concentration in the medium decreased to 4.79–24.28 mg L^−1^ (Fig. 2A). Under the condition of 10 °C, the nitrogen concentration in the medium of 3 mM and 9 mM nitrogen treatments decreased rapidly, and the uptake was basically complete after 12 days of culture. The nitrogen concentration in the 18 mM nitrogen treatment was also absorbed rapidly and decreased to 4.19 mg L^−1^ after 15 days of culture (Fig. 2B). Under the condition of 15, 20, and 25 °C, the nitrogen concentration in the medium was completely absorbed after different days of cultivation, respectively (Fig. 2C–E). Under the condition of 27 °C, the nitrogen concentration in the medium decreased to 4.84–18.51 mg L^−1^ on the 15th day (Fig. 2F). Under 30 °C culture condition, the nitrogen concentration decreased to 7.46–155.71 mg L^−1^ on the 6th day, even though the cells died after 6 days (Additional file 1: Figure S2D).

The absorption of total phosphorus in the culture medium under different temperatures is presented in Additional file 1: Figure S3. There was a surplus of phosphorus concentration in the medium under all culture conditions. The concentration of phosphorus absorbed by *X. hormidioides* is about 100 mg L^−1^.

### Effects of different temperatures and nitrogen concentrations on lipid accumulation in *X. hormidioides*

**Fig. 3:**
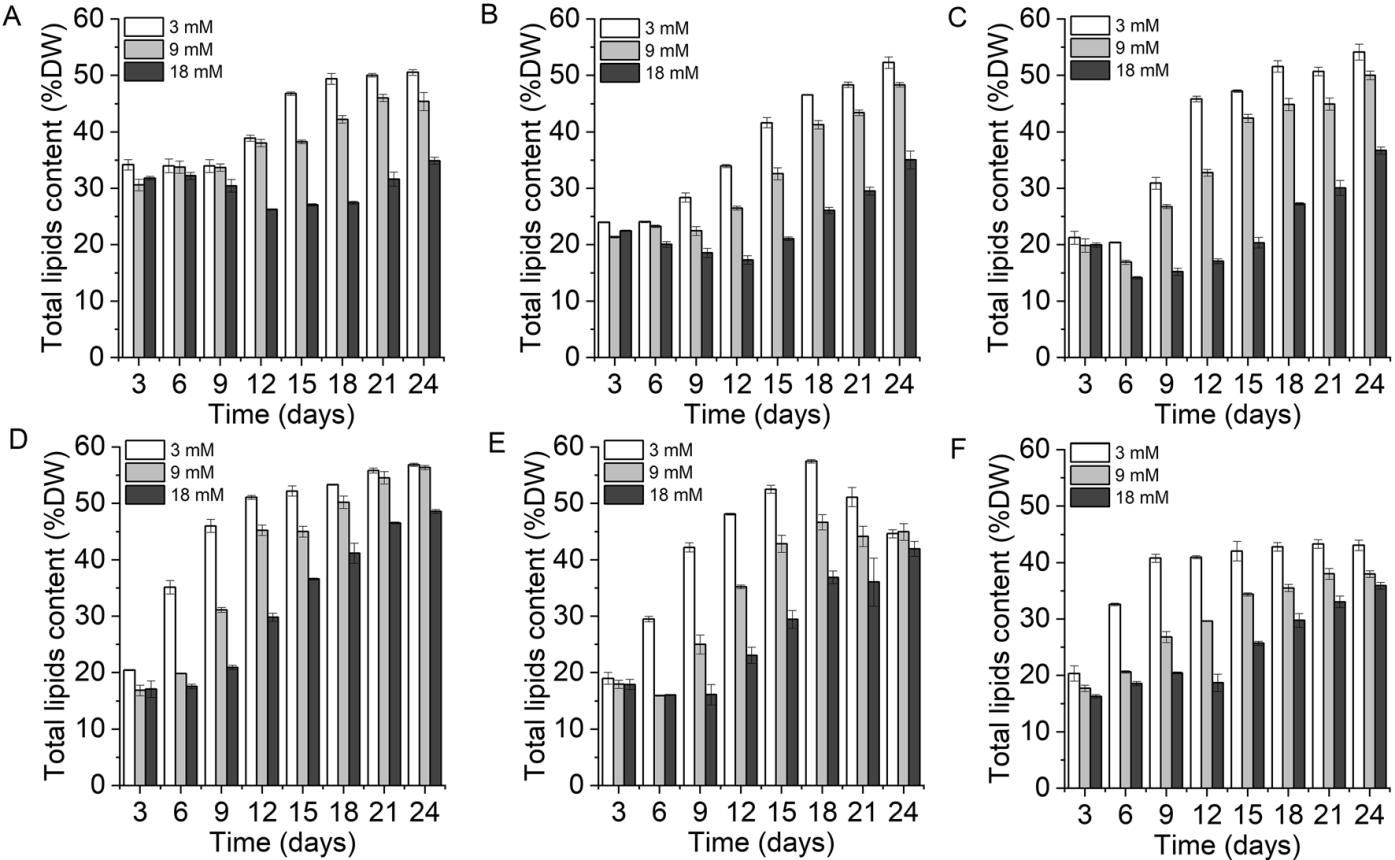
The effect of different temperature on the total lipid accumulation of *Xanthonema hormidioides*; **A**, 7 °C; **B**, 10 °C; **C**, 15 °C; **D**, 20 °C; **E**, 25 °C; **F**, 27 °C; *DW* dry weight

The lipid accumulation of *X. hormidioides* at 8 temperatures was investigated (Fig. 3, Additional file 1: Figure S2). Under different nitrogen treatments, the lipid content decreased with the increase of nitrogen concentration. The highest lipid content was observed at 3 mM nitrogen treatment. At 5 °C, there was little change in the lipid accumulation, but it increased slightly at the later stage of culture (Additional file 1: Figure S2E). When cultured at 7, 10, 15 and 20 °C, lipid accumulation of *X. hormidioides* at the 18 mM nitrogen treatment showed a trend of decreasing first and then increasing with time, and time points of lipid increase were consistent with the adaptation time for growth under each temperature treatment, which were 13, 7, 5 and 3 days, respectively. The highest lipid content was obtained at the end of the culture, and it was 50.54–56.83% of dry weight. Microscopic observation of the morphology of *X. hormidioides* also showed distinct oil droplets in the late logarithmic stage of the cells (Additional file 1: Figure S1). When cultured at 25 °C with 3 mM nitrogen treatment, the lipid content decreased after cultivation of 18 days. The highest lipid content was 57.49% of dry weight achieved at day 18. At 27 °C, the maximum lipid content appeared on the 21st day of the 3 mM nitrogen treatment, which was 43.27% of dry weight. When cultured at 30 °C, the cells gradually died and the lipid content decreased (Additional file 1: Figure S2F).

### Effects of different temperature and nitrogen concentrations on the fatty acid compositions and contents of *X. hormidioides*

**Fig. 4:**
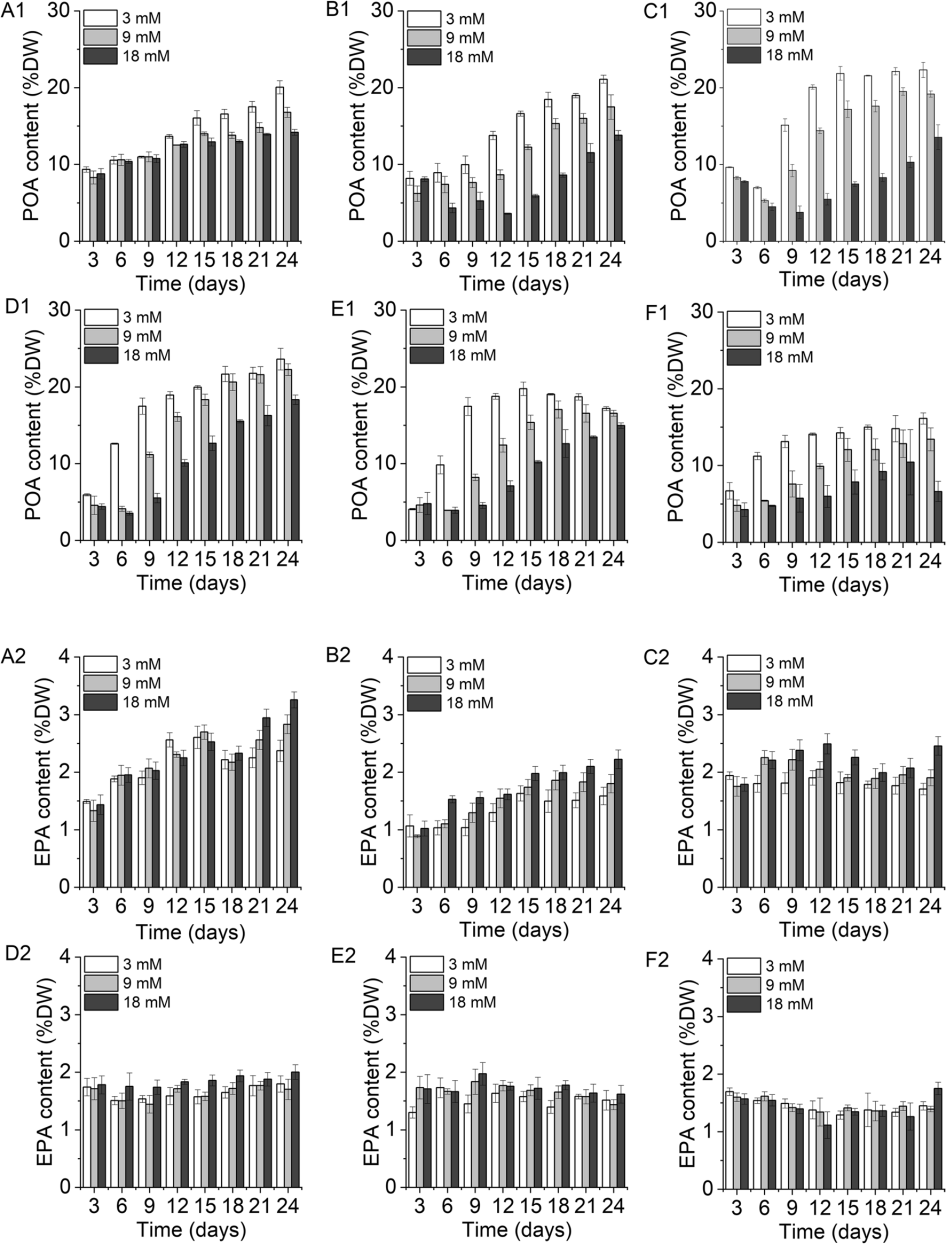
Palmitoleic acid (POA) and eicosapentaenoic acid (EPA) contents of *X. hormidioides* in terms of dry weight under different nitrogen concentrations and temperatures (the three columns of each time point show different nitrogen concentration treatment, from left to right as 3, 9 and 18 mM nitrogen treatment, respectively; **A**, 7 °C; **B**, 10 °C; **C**, 15 °C; **D**, 20 °C; **E**, 25 °C; **F**; 27 °C)

The main fatty acids of *X. hormidioides* were myristic acid (C14:0, MA), palmitic acid (C16:0, PA), palmitoleic acid (C16:1ɷ7, POA), linoleic acid (C18:2ɷ6, LA), eicosatrienoic acid (C20:3ɷ6, EEA), and eicosapentaenoic acid (C20:5ɷ3, EPA), and contained small amounts of γ-linolenic acid (C18:3ɷ6) and arachidonic acid (C20:4ɷ6). The change trend of TFAs content (% of dry weight) with time at different temperatures and nitrogen concentrations was similar to that of lipid (Additional file 1: Figure S4). The change pattern of individual fatty acid in total fatty acids (TFAs) with time under different nitrogen concentrations and temperatures is presented in Additional file 1: Figure S5. The content of POA, the most abundant fatty acid in *X. hormidioides*, gradually increased with increasing culture time and was higher in the low nitrogen treatments (Fig. 4A1-F1). The highest relative content of POA at each temperature was more than 53% of TFAs, and the highest absolute content of POA at each temperature was 16.13% (27 °C)–23.64% (20 °C) of dry weight. The changing trend of EPA content in *X. hormidioides* with culture time was not consistent at each temperature (Fig. 4A2-F2). Under the culture condition of 7 °C, the EPA content increased with culture time, and the highest content was obtained on the last day of culture in 18 mM nitrogen treatment, which was 3.26% of dry weight (Fig. 4A2). At 10 °C, 15 °C, and 20 °C, the relative content of EPA showed a trend of increasing first and then decreasing with time, and the highest content of 19.35%, 23.25% and 21.59% of TFAs was achieved at the 12th, 9th and 6th day, respectively (Additional file 1: Figure S5). The highest absolute content of EPA was more than 2.00% of dry weight (Fig. 4B2-D2). Under the conditions of 25 °C and 27 °C, the highest contents were 1.91% and 1.70% of dry weight, respectively (Fig. 4E2, F2).

### The metabolism in response to variation of temperature and nitrogen concentration in *X. hormidioides* through proteomic analysis

**Fig. 5:**
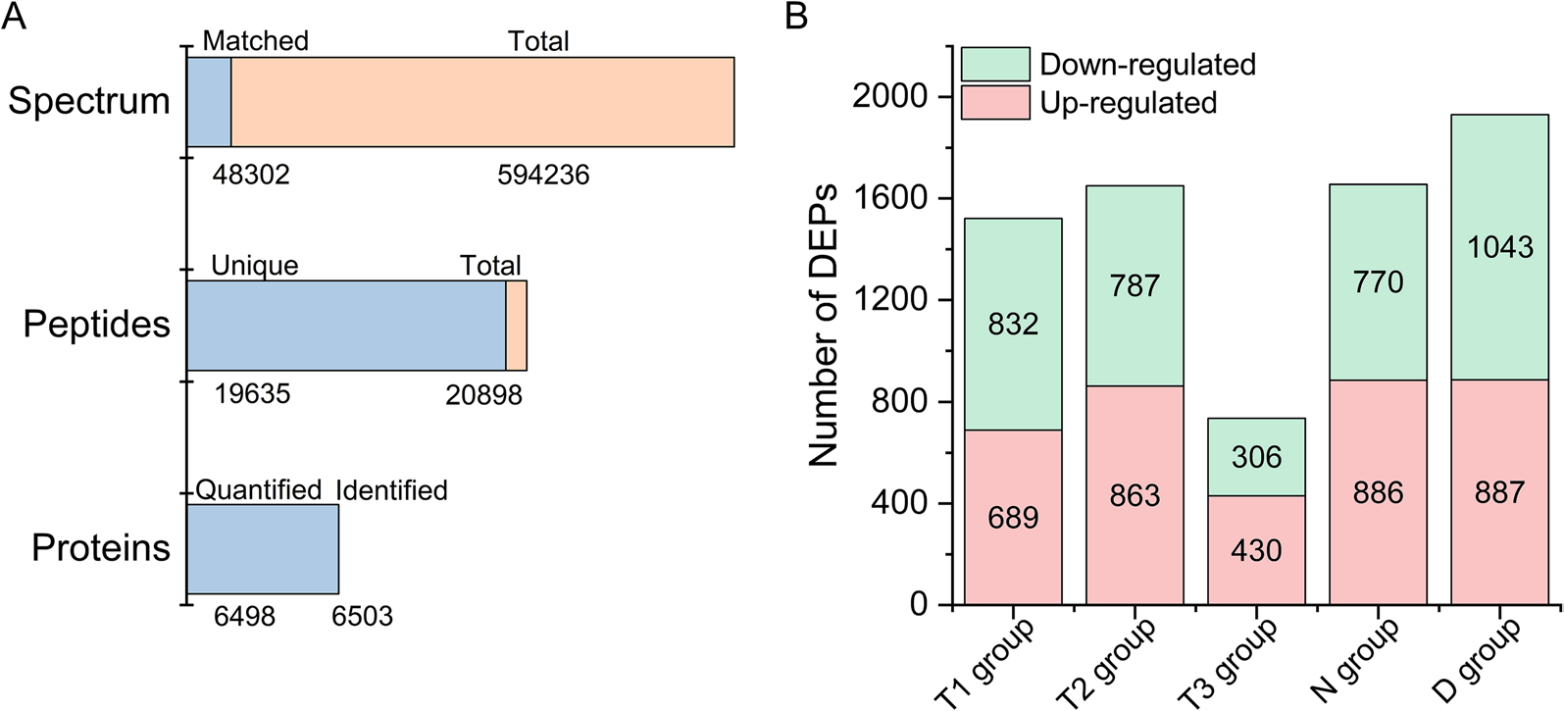
Protein identification information statistics (**A)** and differentially expressed protein (DEPs) identified among different comparison groups (**B)**; T1 group, T7 vs T25; T2, T15 vs T25; T3, T15 vs T7; N, T15-LN vs T15; D, T7 vs T7-d3

In order to thoroughly investigate the effects of temperature and nitrogen concentration treatment on the metabolism of *X. hormidioides*, and decipher the response mechanism of low temperature and low nitrogen concentration, TMT (tandem mass tags) technique was used to identify and quantify proteins for *X. hormidioides* under different temperatures, nitrogen treatments as well as cultivation time points. Protein samples were obtained from the algal cultures grown at temperature of 7 °C with 18 mM nitrogen treatment for 3 days (T7-d3) and 12 days (T7), and the algal cultures grown at temperature of 15 °C for 12 days with 18 mM nitrogen treatment (T15) and 3 mM nitrogen treatment (T15-LN), and the algal cultures grown at 25 °C with 18 mM nitrogen treatment for 12 days (T25). Each protein sample has three biological replicates. Overall, a total of 594,236 spectra were identified, of which 48,302 spectra were matched with the available spectra in the database (Fig. 5A). The number of total peptide and unique peptide sequences was 20,898 and 19,635, respectively. 6503 proteins were finally identified, of which 6498 proteins were quantified.

Five groups of samples, which were T1 (T7 vs T25), T2 (T15 vs T25), T3 (T15 vs T7), N (T15-LN vs T15) and D (T7 vs T7-d3), were compared to identify the differentially expressed proteins (DEPs) (Fig. 5B). For the comparisons of different temperature, the number of DEPs in the group of T1 and T2 were significantly higher than that of T3. A total of 1,521 and 1,650 DEPs were identified as significantly affected in T1 and T2 group, respectively. In the T3 group, 58% of DEPs were up-regulated and 42% were down-regulated. For the comparison of different nitrogen treatment, a total of 1,656 DEPs were identified between T15-LN and T15, of which 54% were up-regulated and 46% were down-regulated. The most numbers of DEPs were revealed in the D group, both the number of up-regulated and down-regulated DEPs increased as the treatment time increased from day 3 to day 12.

The DEPs identified in five groups of comparative samples were performed the enrichment analysis based on GO and KEGG database. The results of GO analysis showed that most of DEPs were annotated as cellular process and metabolic process of the category of biological process, binding and catalytic activity of the category of molecular function, cell part and cell of the category of cellular component (Additional file 1: Figure S6). KEGG enrichment analysis showed that proteins involved in the pathway of ribosome (level 2: translation), photosynthesis (level 2: energy metabolism), purine metabolism (level 2: nucleotide metabolism), glycolysis/gluconeogenesis (carbohydrate metabolism), and lipid metabolism were most affected in treatments (Additional file 1: Figure S7).

#### Ribosomal protein

The ribosome is the site of protein synthesis in the cell, consisting of a large subunit and a small subunit combined with RNA. The protein subunit of the ribosome can maintain the stability of RNA configuration, and its function is mainly to catalyze the formation of peptide bonds in the peptide chain extension stage and the hydrolysis of ester bonds in the termination stage. The results showed that under comparison of T1, the protein abundances of 49 large protein subunits and 31 small protein subunits were significantly up-regulated in low temperature of 7 °C, and only 1 small protein subunit (L6) was down-regulated (Additional file 1: Figure S8a). At T2, the protein abundances of 33 large protein subunits and 21 small protein subunits were significantly increased under 15 °C, and 6 protein subunits were down-regulated (Additional file 1: Figure S8b). Under low nitrogen stress (N), there were 30 protein subunits up-regulated, and 18 protein subunits down-regulated (Additional file 1: Figure S8c). Compared to the culture of 7 °C of day 3, the protein abundances of 27 large protein subunits and 21 small protein subunits were significantly increased, and 18 large protein subunits and 13 small protein subunits were down-regulated of day 12 (Additional file 1: Figure S8d). Many DEPs were involved in the pathway of ribosome indicated proteins biosynthesis and degradation were important for adaptation to low temperatures and low nitrogen.

#### Photosynthesis

Photosynthetic electron transport is driven by the absorption of light energy, which flows through photosystem II (PSII), cytochrome *b6f* and photosystem I (PSI). NADPH is the product of electron transfer, and ATP is produced by chloroplast ATP synthase using the proton gradient established across the thylakoid membrane during electron transport [13]. Photosynthesis-related DEPs are presented in the Additional file 1: Figure S9. At T1 group, compared to the culture under 25 °C, the protein abundances of PsbA (D1) and PsbD (D2) of photosystem II, PsaB, PsaD and PsaF of photosystem I and PetH (FNR) of photosynthetic electron transport were down-regulated in the low temperature of 7 °C. Only one DEP of PetF (Fd) was up-regulated (Additional file 1: Figure S9a). At T2 group, all 10 DEPs including PsbB (cp47), PsbO (MSP), PsbQ of photosystem II, PetB and PetA of cytochrome b6/f complex, PetF and PetH of photosynthetic electron transport and beta, alpha and b subunit of ATPase were up-regulated (Additional file 1: Figure S9b). At N group, 9 DEPs of photosystem II, photosystem I, photosynthetic electron transport and ATPase were down-regulated. Only one DEP of PsbU was up-regulated (Additional file 1: Figure S9c). At D group, 12 DEPs of photosystem II, cytochrome b6/f complex, photosynthetic electron transport and ATPase were up-regulated. Two DEPs of PsbA and PsbD was down-regulated (Additional file 1: Figure S9d). The changes of photosynthesis-related proteins were consistent with the growth of Fig. 1.

#### Carbon metabolism

**Fig. 6:**
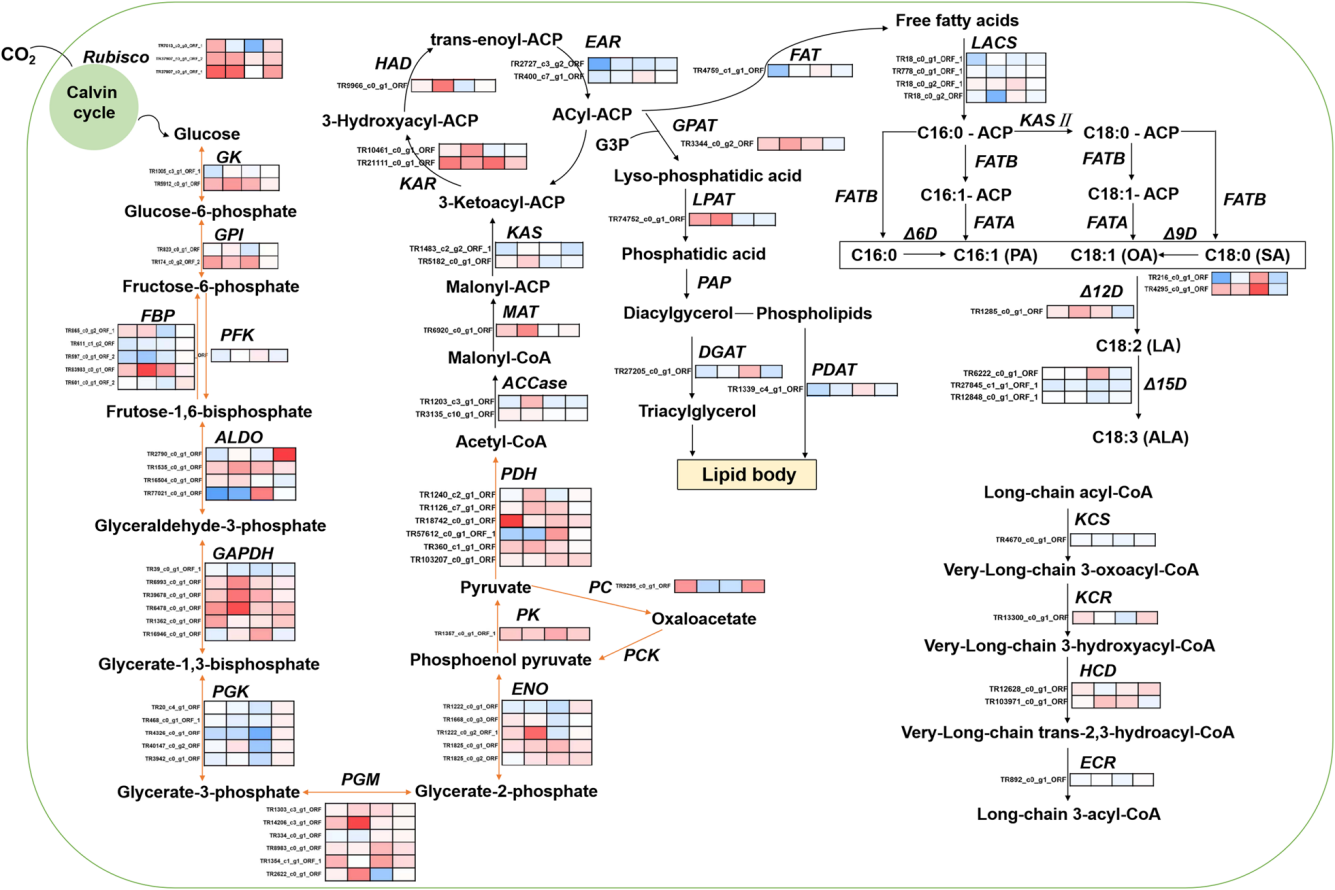
Carbon metabolism; *Rubisco* Ribulose-1,5-bisphosphate carboxylase/oxygenase, *GK* glucokinase, *GPI* glucose-6-phosphate isomerase, *FBP* fructose-1,6-bisphosphatase, *PFK* 6-phosphofructokinase, *ALDO* aldolase, *GAPDH* glyceraldehyde-3-phosphate dehydrogenase, *PGK* Phosphoglycerate kinase, *ENO* enolase, *PK* pyruvate kinase, *PC* pyruvate carboxylase, *PCK* phosphoenolpyruvate carboxykinase, *PDH* pyruvate dehydrogenase, *ACCase* acetyl-coenzyme A carboxylase, *MAT* malonyl-ACP transferase, *KAS* 3-ketoacyl-ACP synthase, *KAR* 3-ketoacyl-ACP reductase, *HAD* 3-ketoacyl-ACP dehydrase, *EAR* 3-enyl-ACP reductase, *G3P* glycerate-3-phosphate, *GPAT* glycerol-3-phosphate acyltransferase, *PAP* phosphatidic acid phosphatase, *DGAT* diacylgycerol acyltransferase, *PDAT* phospholipid: diacylglycerol acyltransferase, *FAT* fatty-acyl-ACP thioesterases, *KCS* 3-ketoacyl-CoA synthase, *KCR* very-long-chain 3-oxoacyl-CoA reductase, *HCD* enoyl-CoA hydratase, *ECR* trans-2-enoyl-CoA reductase

The different pathways related to carbon metabolism showed different trends during cold exposure and low nitrogen treatment. Changes in enzyme abundance are depicted in Fig. 6. Carbon fixation plays an important role in carbon metabolism of microalgae. Photosynthetic carbon fixation, also known as the Calvin cycle, uses ATP and NADPH produced by light reaction to synthesizes organic matter, and promotes the accumulation of biomass and the synthesis of metabolites. Ribulose-1,5-bisphosphate carboxylase/oxygenase (Rubisco) catalyzes the formation of C3 compounds, and the abundance of Rubisco was significantly up-regulated at low temperatures (T1, T2 group) and D group, but it was down-regulated under low nitrogen condition (N group).

The metabolic pathways of glycolysis and gluconeogenesis share most of enzymes for reversible reactions, and utilize different enzymes to catalyze key reactions. 6-phosphofructokinase (PFK) and pyruvate kinase (PK) are key enzymes in the glycolysis pathway [14]. In contrast, the three enzymes that catalyze the irreversible reactions of the gluconeogenesis pathway are fructose 1,6-bisphosphatase (FBP), phosphoenolpyruvate carboxykinase (PCK), and pyruvate carboxylase (PC). The results showed that at low temperature, the protein abundance of PFK maintained at a certain level, and the key enzyme of PK and other enzymes (glucose-6-phosphate isomerase (GPI), fructose-bisphosphate aldolase (ALDO), glyceraldehyde 3-phosphate dehydrogenase (GADPH), phosphoglycerate mutase (PGM), enolase (ENO)) were up-regulated, and the abundance of triosephosphate isomerase (TPI) and phosphoglycerate kinase (PGK) were down-regulated, indicating that the process from glucose to pyruvate was enhanced. At low nitrogen treatment, the protein abundance of FBP, PGK, PC, ALDO and TPI were down-regulated, and the GADPH and PGM were up-regulated, and there were no significant changes in GPI and ENO. The dynamics of these enzymes indicated that the carbon flow directed to carbohydrate accumulation is more active in *X. hormidioides* under high nitrogen concentration.

Pyruvate can be catalyzed by pyruvate dehydrogenase (PDH) to generate acetyl-coenzyme A (acetyl-CoA), which is a precursor for fatty acid synthesis. Microalgal cells synthesize fatty acids in chloroplasts, and acetyl-CoA carboxylase (ACCase) catalyzes the first step and is a rate-limiting enzyme for fatty acid synthesis. The abundance of PDH and ACCase were up-regulated under low temperature culture of T2, and there was no significant difference at T1. In N group, the protein levels of both enzymes increased. Palmitoyl-ACP can be continued to form stearoyl-ACP with acetyl-CoA, or palmitoylthioesterase can be used to synthesize palmitic acid, which is released free from the fatty acid synthase complex [15]. The product can further form long-chain saturated fatty acids and polyunsaturated fatty acids through the action of carbon chain elongase and fatty acid desaturase and dehydrogenase [16]. TAG synthesis includes the acetyl-CoA dependent pathway, also known as the Kennedy pathway, and the acetyl-CoA independent pathway. For the Kennedy pathway, acetyl-CoA is sequentially added to the sn-1, sn-2, and sn-3 positions of 3-phosphoglycerol (G3P) through the catalysis of glycerol-3-phosphate acyltransferase (GPAT), lysophospholipid acyltransferase (LPAT), and diacylglycerol acyltransferase (DGAT). DGAT is the rate-limiting enzyme in TAG biosynthesis [17]. The acetyl-CoA independent pathway for TAG synthesis is catalyzed by phospholipid:diacylglycerol acyltransferase (PDAT). Both glycerol-3-phosphate acyltransferase (GPAT) and lysophospholipid acyltransferase (LPAT) were significantly up-regulated in low temperature culture (T1, T2). While, the key enzyme DGAT and PDAT were only up-regulated under N group, but not under T1, T2 and D group. The results indicated that pyruvate mainly supplied TAG accumulation under low nitrogen treatment. It was consistent with the result of lipid content (Fig. 3). The lipid content on the 12th day of the culture at 7 °C, 15 °C, and 25 °C was about 20–25% of dry weight, and there is no significant difference, but the lipid content was 2.68 times higher at low nitrogen concentration than at high nitrogen concentration.

Fatty-acyl-ACP thioesterases (FAT) catalyze the hydrolysis of acyl-ACP to form free fatty acids, which undergo carbon chain elongation, desaturation and esterification reactions in endoplasmic reticulum [18]. There are two classes of thioesterases, FATA and FATB, which are responsible for the hydrolysis of unsaturated and saturated acyl-ACP, respectively [19]. Desaturases are responsible for the insertion of double bonds into specific carbon positions of fatty acids, and differential regulation of desaturases indicates that the position of the double bond is tightly controlled [20]. Unfortunately, not many fatty acid desaturases have been identified through the proteomics of *X. hormidioides*. The abundance of desaturases identified showed that delt-9 desaturase (Δ6D) and delt-12 desaturase (Δ12D) were up-regulated in T1, T2 and N group, while delt-15 desaturase (Δ15D) did not change significantly.

#### Signal transduction-related proteins

Signal transduction helps cells sense the presence of stress factors in the external environment and transmit them in the form of signals to form a certain self-defense system and regulate the expression of the corresponding proteins. Phosphatidylinositol signaling system is a signal network closely related to calcium signaling, which can not only affect gene expression, but also participate in cell response to the change of external environment [21]. In this system, protein kinase C (PKC) and phospholipase C (PLC) play a critical role in the regulation of the pathway and the activation of related genes [22]. In *Chlamydomonas reinhardtii*, some of these PKs were significantly induced or repressed by cold stress [23]. Meanwhile, some literatures have shown that the expression of inositol monophosphatase (IMP) gene can be induced by stress, and it has a certain anti-stress function [24]. In this experiment, it was found that the protein levels of calcium binding protein (CaBP) and IMP were significantly increased under low temperature condition, and the protein abundance of PKC, PLC and IMP were significantly up-regulated under T1 and T2 (Table 1). The protein levels of PKC and PLC were increased in D group, indicating that *X. hormidioides* has a complex signaling pathway to eliminate the external damage to cells under stress.

**Table 1:** Protein involved in phosphatidylinositol signal system

Enzyme	T_1_	T_2_	N	D
PKC	↓	↓	↑	↑
PLC	↑	↓	↑	↑
CaBP	↑	↑	↓	↓
IMP	↑	↑	↑	↓

#### Antioxidant-related proteins

**Fig. 7:**
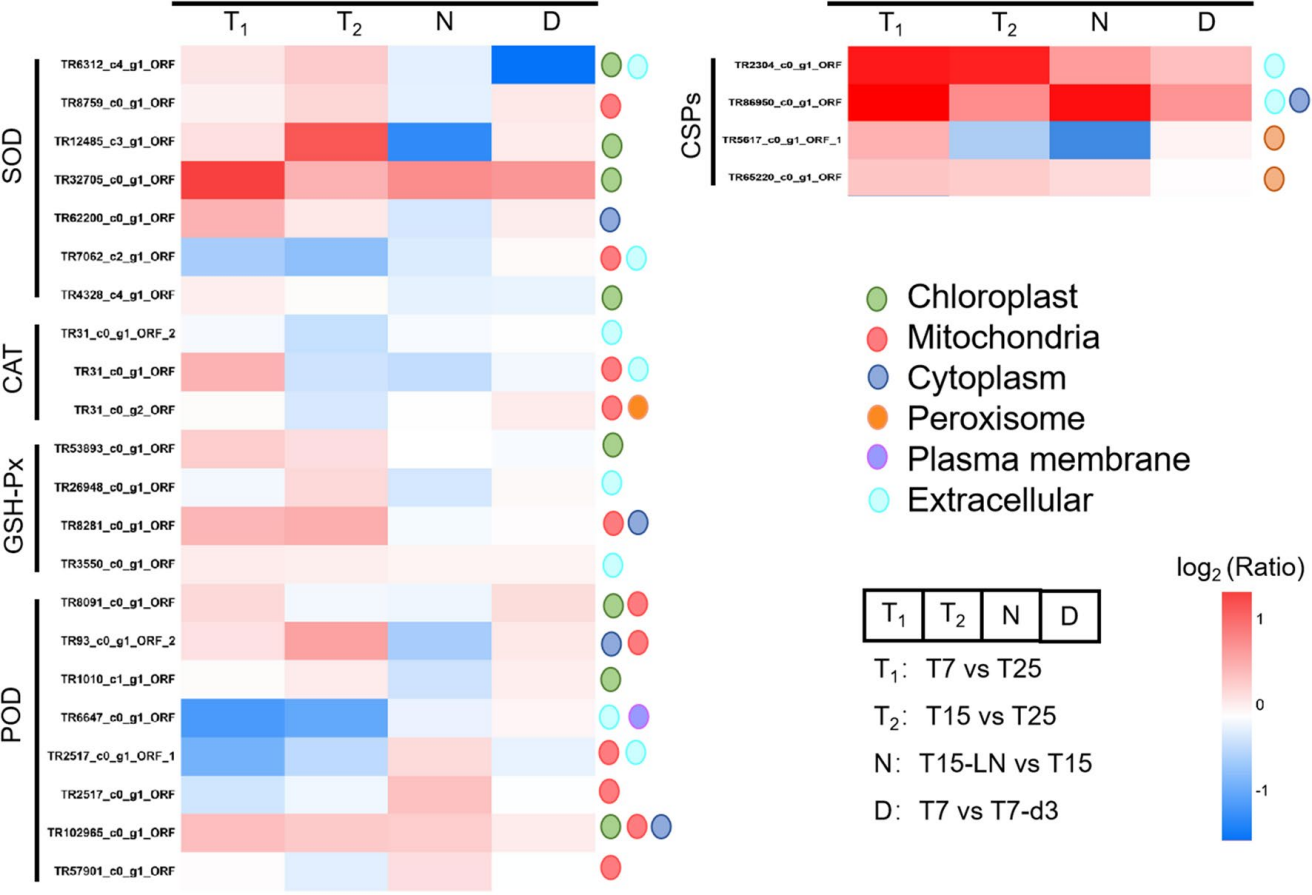
Heatmap of antioxidant related proteins and cold shock proteins

Stress conditions may lead to energy imbalance which induces the generation of reactive oxygen species (ROS), and high levels of ROS attack cell membranes and damage cells [13]. Cells can produce antioxidants like carotenoids, ascorbic acid, glutathione, and antioxidant enzymes such as superoxide dismutase (SOD), catalase (CAT), glutathione peroxidase (GPX), ascorbate peroxidase (APX), peroxidase (POD), etc., to scavenge ROS and to protect cells [25]. The results showed that the protein abundance of SOD and GPX increased at low temperature in *X. hormidioides* (Fig. 7). While, some of SOD and POD were more active at low nitrogen concentration treatment. The antioxidant system-related enzymes were up-regulated under low temperature stress, indicating that the synergistic effect of these protective enzymes alleviated the oxidative damage caused by low temperature stress, and reached a new balance with the metabolic activities to guarantee the growth and metabolism of the cells, and improved its temperature adaptability.

#### Cold shock proteins

Cold shock proteins (CSPs) are produced by cells in response to cold stimuli. They are structurally rich in aromatic amino acids and play an important role as molecular chaperones in enhancing the ability of cells to withstand cold stress. As an RNA chaperone, it can maintain membrane fluidity, stabilize the secondary structure of nucleic acid, and improve the efficiency of transcription and translation, etc. [26]. At 7 °C and 15 °C, the level of CSPs in *X. hormidioides* increased significantly (Fig. 7), indicating that these proteins could reduce the damage caused by low temperature stress, and maintain the normal growth and metabolism of the cells.

## Discussion

Temperature is an important growth-determining factor for microalgae, and the effect of temperature on the microalgal growth could be described as a bell-shaped curve, with a gradual increase in growth rate below the optimal temperature and a sharp decrease above the optimal temperature. According to the minimum temperature (T_min_), optimum temperature (T_opt_), and maximum temperatures (T_max_) for growth, cold-adapted microalgae include psychrophilic microalgae (T_min_ ≤ 0 °C, T_opt_ ≤ 15 °C and T_max_ = 15–20 °C) and psychrotrophic or psychrotolerant microalgae (T_min_ > 0 °C and T_opt_ > 15 °C) [5]. In our study, compared with the room temperature of 25 °C, the growth of *X. hormidioides* at 10 °C, 15 °C and 20 °C was much higher, and the highest biomass yield was 1.02, 1.60 and 1.70 folds that of 25 °C, respectively. The optimum temperature range for the growth of *X. hormidioides* was between 15 and 20 °C, and the growth was very slow at 5 °C, and the cells died when the temperature reached 30 °C. When we cultivated the algae seed at 7 °C for a month, it could quickly grow at 5 °C, and the final biomass could reach 2.7 g L^−1^ (data not shown). Meanwhile, we tried to freeze this algae culture suspension in the refrigerator at −20 °C, and found that it slowly turned green after 3 months, which proved that this algae can survive and recover at temperature below 0 °C as well. It can be determined that *X. hormidioides* is a psychrotrophic or psychrotolerant microalga. Too low temperature (less than 5 °C) inhibited the growth of *X. hormidioides*, and high temperature (≥ 30°C) caused the death of *X. hormidioides*. Depending on the purpose, an appropriate temperature for microalgae cultivation can be selected, but as with other factors, the optimal temperature varies for each microalga [2]. Microalgae are usually grown in a temperature range of 15–30 °C, with an optimum temperature of 20–25 °C [3, 27]. *Nannochloropsis limnetica* can grow in the range of 15–27 °C, and maximum biomass is obtained at 22 °C [28]. The optimum growth temperatures for *Tetraselmis subcordiformis* and *N. oculata* are 20 °C and 25 °C, respectively [29], and the optimum temperature range for the growth of of *Chlorella* sp., *Nannochloropsis* sp., *Chlamydomonas* sp., *Botryococcus* sp., *Scenedesmus* sp., *Neochloris* sp., *Ulva* sp. and *Haematococcus* sp. were reported to be 20–30 °C [30]. Nitrogen is an important macro-element that constitutes the biochemical composition of microalgal cells, including nucleic acids (DNA, RNA), proteins, pigments, etc. Its concentration has a significant impact on the growth of microalgae. The growth of *X. hormidioides* was increasing with nitrogen concentration ranging from 3 to 18 mM. According to previous report, the affinity for nitrogen uptake is dependent on temperature, and the uptake rate decreases at lower temperatures, thereby inhibiting the growth rate [31, 32]. The results of nitrogen uptake rate by *X. hormidioides* at different temperatures also confirmed this conclusion.

Microalgal lipid productivity and fatty acid composition vary with temperature, as biochemical pathways related to lipid synthesis and accumulation are controlled by enzymes that are highly sensitive to thermal changes [2]. For our study on *X. hormidioides*, the lipid content at different nitrogen concentrations showed different trends over time at different temperatures, and the reason for this difference may be related to the rate of nitrogen uptake by *X. hormidioides* at different temperatures, more precisely, it is related to the nitrogen stress in the cell. Nitrogen limitation is the most widely used strategy to stimulate lipid accumulation in microalgae [3]. According to the variation of medium nitrogen concentration (Fig. 2), *X. hormidioides* faced different times of nitrogen stress under different temperature and nitrogen concentration treatment. Among them, at 5 °C, the nitrogen in the culture medium had not been completely absorbed, so the cells were in nitrogen sufficiency and its lipid content did not change too significantly over time. In nitrogen-limited media, CO_2_ fixed by the cells is preferentially converted to energy storage substances such as lipids and carbohydrates, and only a small amount of available nitrogen is used to synthesize enzymes and cellular structures necessary for cell growth. For oleaginous microalgae, nitrogen stress conditions lead to reduced cell division, thereby promoting the lipid biosynthesis pathway to synthesize more neutral lipids than membrane lipids required for cell wall composition [33]. Under the nitrogen concentration of 3 mM, the lipid content accumulated by *X. hormidioides* can reach 56.6% of the dry cell weight, indicating that low nitrogen can effectively promote the lipid accumulation of *X. hormidioides*. The protein abundance of enzymes involved in lipid synthesis also demonstrated the accumulation of lipids under low nitrogen treatment, especially the up-regulation of key enzymes DGAT and PDAT. In eukaryotes, energy can be stored as triacylglycerol (TAG), which is an essential carbon source and an important component that contributes to survival under harsh environmental conditions [25]. Most microalgae accumulate triacylglycerols under conditions of nitrogen or phosphorus limitation, high salinity, and temperature stress [34–36]. Comparison of current study with previous reports of microalgae under low temperature, the highest biomass, lipid and fatty acids content of *X. hormidioides* was higher than other microalgae (Table 2).

**Table 2:** Comparison of *X. hormidioides* with other cold-adapted microalgae

Species	Culture apparatus	Temperature (°C)	Biomass (g L^−1^)	Lipid content	Fatty acids content	References
*Acutodesmus obliquus* MR	1 L PBR	10, 25	1.61	42.30%	nd	[32]
*Tetrasemis chuii* SAG 1.96	310 mL PBR	8, 15	5.82	nm	6.40%	[1]
*Pseudopleurochloris antarctica*	310 mL PBR	8, 15	3.30	nm	34.40%	[1]
*Chlamydomonas* sp. RCC 2488	310 mL PBR	8, 15	3.39	nm	36.05%	[1]
*Koliella antarctica* SAG 2030	380 mL PBR	15	11.68	nm	27.19%	[37]
*Chlorella vulgaris* ABC-002	500 mL Erlenmeyer flasks	10, 25	3.40	27.95%	nm	[38]
*Chlamydomonas pulsatilla*	350 mL PBR	6	3.37	39.20%	nm	[39]
*Xanthonema hormidioides*	1.3 L PBR	7, 10, 15, 20, 25, 27	11.73	56.63%	41.14%	This study

*PBR* photobioreactor, *nm* not measured

Under stress conditions, cells show an increase in fatty acid synthesis. The total fatty acid content and palmitoleic acid content accumulated by *X. hormidioides* under low nitrogen conditions were higher than those under high nitrogen conditions, which was related to the accumulation of lipids. At low temperatures, the cell membrane fluidity of microalgae is weakened, which affects the function of enzymes and proteins on the membrane. Synthesizing more unsaturated fatty acids can reduce the rigidity of microalgal cell membrane lipids at low temperatures, thus maintaining membrane fluidity [40]. Among the enzymes involved in fatty acids metabolism, ∆9 fatty acid desaturase catalyzes the first step of desaturation and initiates the conversion of saturated fatty acids to monounsaturated fatty acids, which is essential for the production of polyunsaturated fatty acids [25, 41]. Several fatty acid desaturases were up-regulated in Antarctic ice alga *Chlamydomonas* sp. ICE-L when it was under low temperatures [20]. *Chlorella zofingiensis* substantially up-regulated the transcripts of ∆9 fatty acid desaturase under stress conditions such as high light and nitrogen deficiency, and resulted in the accumulation of total fatty acids, including oleic acid [42]. For the proteomic analysis of *X. hormidioides*, up-regulation of ∆9 fatty acid desaturase was observed under low temperatures and low nitrogen treatment. The most common change in response to low temperature was an increase of unsaturated fatty acids content, but an increase of short chain, methyl branched and/or cis-isomeric fatty acids content were also common [43, 44].

Algae regulate the photosynthetic machinery to adapt to stress conditions. Photosynthesis is a temperature-sensitive energy conversion process that converts light energy into chemical energy. Exposure to stress condition will cause an inequity between energy consumption and supply in photosynthetic algae, leading to alterations to the photosynthetic apparatus [25]. Low-temperature stress affects many aspects of photosynthesis in algae [4]. In *Chlamydomonas*, some of the light harvesting complex proteins and *b6f* cytochromes decline after temperature reduction, leading to a decrease in photosynthetic capacity [40]. It was also observed in *X. hormidioides* at low temperature of T1 group and N group, which the proteins of PSII, PSI, photosynthetic electron transport and F-type ATPase were down-regulation. The down-regulation of photochemical processes facilitates the dissipation of excess energy to protect the photosynthetic apparatus at low temperatures [45]. Interestingly, several studies showed that the expression of genes associated with photosystems increases at low temperature in Antarctica microalgae [46]. For example, the expression of PSII core proteins encoding genes (psbA and psbC) in the polar diatom *Fragilariopsis cylindrus* was up-regulated during low temperature acclimation [47]. For *X. hormidioides*, the proteins related to photosystems was up-regulated at 15 °C of T2 group and D group. It may be a reflection in the low temperature adaptation of *X. hormidioides*. Key enzymes associated with CO_2_ fixation, such as Rubisco, play a critical role in photosynthesis [25]. The expression level of Rubisco is significantly affected by various stresses. Deng et al. [48] reported that rbcL encoding the large subunit of Rubisco was up-regulated at elevated temperatures up to 20 °C in green alga *Chaetomorpha valida*, but its expression was down-regulated with further increases in temperature. In *X. hormidioides*, the abundance of Rubisco was significantly up-regulated at low temperatures thereby increasing carbon fixation.

Photosynthesis creates an aerobic environment within the cell, which is exacerbated by low temperatures and other stress-induced metabolic imbalances that lead to the formation of ROS, and this is further amplified by increased oxygen solubility at low temperatures, resulting in a hyperoxic extracellular environment [26]. ROS elevation induces the synthesis of ROS scavenging enzymes. As a first line of defense, SOD rapidly converts superoxide (O_2_^−^) to O_2_ and H_2_O_2_, and the resulting H_2_O_2_ can be converted to H_2_O by peroxidases such as APX and GPX found in vesicles, cell walls, and cytosol [25]. *Chlorella ellipsoidea* was reported to produce high levels of SOD in response to temperature stress [49]. In addition, CAT is considered as the dominant enzyme that catalyzes the exchange of H_2_O_2_ with H_2_O or other non-toxic molecules [25]. The sea-ice diatom *Entomoneis kufferathii* increased activity of CAT after exposure to high light and low temperature, which help protect cells from oxidative damage [50]. For *X. hormidioides* in our study, the up-regulation of SOD, CAT, GPX, and POD indicated that it had highly effective antioxidant systems under stress condition including low temperature and low nitrogen.

Protein synthesis is a very important step for growth in the low temperature. The up-regulated of ribosomal proteins at low temperature treatment (T1, T2 group) of *X. hormidioides* suggests that protein synthesis is increased to reduce the damage caused by low temperature stress. Protein synthesis might be a rate-limiting factor in the induction of compensatory cellular responses in cold [51]. The antarctic bacterium *Pseudoalteromonas haloplanktis* has been shown to synthesize proteins with 30% up-regulation even at 4 °C [52]. Same phenomenon of protein over-expression in link with translation have also been reported in different cold-adapted bacteria [51]. Protein synthesis requires corresponding cofactors, among which EF-TU plays an important role in the extension stage of peptide chain [53]. Several EF-Tu proteins were significantly increased at low temperature (Additional file 1: Figure S6a, b), indicating that EF-Tu proteins played a positive regulatory role in cold adaption. EF-Tu also reduces the aggregation of various protein residues under environmental stress until the stress disappears and these proteins return to normal, protecting cells from the inability of growth and metabolic activities caused by permanent denaturation of proteins [53]. In conclusion, the expression of ribosomal protein, EF-TU was up-regulated under low temperature treatment, which not only promoted the synthesis of new protein, but also slowed down the protein denaturation, and finally reduced the damage brought by low temperature stress to cells. For N and D group, the decrease of ribosomal proteins at low nitrogen treatment (N group) suggests that protein synthesis is decreased at low nitrogen concentration to conserve energy.

Psychrophiles and psychrotrophs can counteract temperature changes by synthesizing CSPs [51]. CSPs are small molecular weight proteins, single-stranded, and an important feature of cold-adapted microorganisms. They bind to nucleic acids and help regulate a variety of cellular processes, such as protein folding, transcription, translation and membrane fluidity [6, 54]. Zhang et al. [55] reported that CSPs mentors nascent mRNAs to develop secondary structures for efficient protein expression. In our research, the level of CSPs protein increased significantly at low temperatures. CSP is induced by several microorganisms at low temperatures, including *Psychrobacter cryohalolentis* K5 and *Shewanella livingstonensis* Ac10 [56]. CSPs were abundant in polar diatom *Fragilariopsis cylindrus* and were significantly elevated after the transition from 4 °C to 0 °C [26].

## Conclusion

*X. hormidioides* is a robust microalga that can grow over a wide range of temperatures. It was able to grow from 5 to 27 °C, and grows best between 15 and 20 °C, indicating that it is a psychrotrophic microalga. The highest biomass occurred at the initial high nitrogen concentration (18 mM), which was 11.73 g L^−1^ at 20 °C. Under the combined effect of temperature and nitrogen concentration, the highest lipid, total fatty acids and POA content accumulated by *X. hormidioides* was 56.83%, 41.14% and 29.09% of dry weight, respectively. *X. hormidioides* is a highly lipid and palmitoleic acid producing microalga with great potential for biofuel development, as well as for applications in nutritional health products and other industries. Meanwhile, the TMT quantitative proteome was used to analyze the protein levels of *X. hormidioides* under different temperatures and nitrogen supply, revealing the internal regulatory mechanisms of proteins in physiological changes induced by temperature and nitrogen stresses. At low temperature and low nitrogen culture, the proteins of PSII, PSI, photosynthetic electron transport and F-type ATPase were down-regulation to protect the photosynthetic apparatus. The levels of key enzymes of the Calvin cycle (Rubisco, GAPDH) and glycolytic pathway (PK) were elevated under low temperature, leading to pyruvate accumulation, and pyruvate production of acetyl CoA was enhanced. The levels of ACCase, GPAT and LPAT proteins were elevated, thereby promoting intracellular carbon flow to fatty acid synthesis. The up-regulation of key enzymes DGAT and PDAT demonstrated the accumulation of TAGs under low nitrogen treatment. The proteins related to ribosome, phosphatidylinositol signaling system, antioxidants and CSPs were increased to guarantee the growth and metabolism of the cells under low temperatures.

## Material and methods

### Cultivation condition

*Xanthonema hormidioides* 836-1 was obtained from the Culture Collection of Algae at the University of Göttingen (SAG). It maintained in a conical flask containing 50 mL modified Endo medium without glucose [57]. Then it was transferred to a glass columnar photobioreactor (Ø6.0 × 60 cm) with working volume of 1.3 L as algal seeds.

The algal seeds were growing to the logarithmic stage, and then the original medium was removed. Three initial nitrogen concentrations of 3, 9 and 18 mM, and 8 temperature gradients of 5, 7, 10, 15, 20, 25, 27, 30 °C, were investigated in this study. Each culture condition set three parallel groups. The initial inoculation biomass concentration was 0.3 ± 0.03 g L^−1^. All cultures were aerated continuously with compressed air containing 1% CO_2_. The light intensity was 300 μmol·photons·m^−2^ s^−1^ for 24 h. The schematic diagram of the cultivation device is shown in the Additional file 1: Figure S8.

### Analysis method

*Determination of biomass, N and P concentrations, lipid content and fatty acids*: 10 mL of algal culture suspension was taken every day to measure biomass, N and P concentrations in the medium, and 80 mL of algal culture suspension was collected every 3 days to harvest algal powder. The measurement method of biomass, lipid content, fatty acids were according to Gao et al. [57]. The supernatant of N and P concentrations was measured on an AutoAnalyzer3 instrument (Bran-Luebbe, Germany).

*Proteomic analysis*: Algal samples lysis and protein extraction were performed using SDT (4% SDS, 100 mM Tris–HCl, 1 mM DTT, pH 7.6) method [58]. The protein was quantified with the BCA Protein Assay Kit (Bio-Rad, USA), and was then digested by trypsin according to Matthias Mann [59]. The digest peptides of each sample were desalted on C18 Cartridges, and labeled using Tandem Mass Tags (TMT) according to the manufacturer’s protocol of Thermo Scientific.

Labeled peptides from different culture samples were mixed, and fractionated by High pH Reversed-Phase Peptide Fractionation Kit (Thermo Scientific). For LC–MS/MS analysis, the peptides were loaded onto a reverse phase trap column (Thermo Scientific Acclaim PepMapa100) connected to the C18-reversed phase analytical column (Thermo Scientific Easy Column) via a linear gradient elution of buffer A (0.1% formic acid) and buffer B (84% acetonitrile and 0.1% formic acid) at a flow rate of 300 nL/min. The Q Exactive mass spectrometer (Thermo Scientific) was operated in positive ion mode. MS data were collected using a data-dependent top 10 method to dynamically choose the most abundant precursor ions from the survey scan (300–1800 m/z) for HCD fragmentation.

For protein identification and quantitation analysis, the MS raw data for each sample was performed using the MASCOT 2.2 (Matrix Science, London, UK) embedded into Proteome Discoverer 1.4 software. Differential expression with ratio 1.2 (*P* < 0.05) was defined as up-regulation, that with ratio 0.83 (*P* < 0.05) as down-regulation.

The mass spectrometry proteomics data have been deposited in the ProteomeXchange Consortium via the PRIDE [60] partner repository with the dataset identifier PXD037434.

## Supplementary Information

**Additional file 1: Figure S1. **Cellular morphology of Xanthonema hormidioides; The scale bars indicate 10 μm. **Figure S2.** The effect of three nitrogen concentrations on the growth, consumption of medium nitrogen concentration and lipid content of X. hormidioides under different temperatures; A, C, E, 5 °C; B, D, F, 30 °C. **Figure S3.** The effect of different temperature on the consumption of medium phosphorus concentration; A, 5 °C; B, 7 °C; C, 10 °C; D, 15 °C; E, 20 °C; F; 25 °C; G, 27 °C; H, 30 °C. **Figure S4.** Fatty acid profiles of X. hormidioides in terms of dry weight under different nitrogen concentrations and temperatures (the three columns of each time point show different nitrogen concentration treatment, from left to right as 3, 9 and 18 mM nitrogen treatment, respectively; A, 7 °C; B, 10 °C; C, 15 °C; D, 20 °C; E, 25 °C; F; 27 °C). **Figure S5.** Fatty acid profiles of X. hormidioides in terms of total fatty acids under different nitrogen concentrations and temperatures. **Figure S6.** GO analysis of differentially expressed proteins of X. hormidioides. **Figure S7.** KEGG analysis of differentially expressed proteins of X. hormidioides. **Figure S8.** The expression of ribosomal proteins among different comparison groups; Red, up-regulated; Green, down-regulated; A, T7 vs T25; B, T15 vs T25; C, T15-LN vs T15; D, T7 vs T7-d3. **Figure S9.** The expression of photosynthesis related proteins among different comparison groups; Red, up-regulated; Green, down-regulated; A, T7 vs T25; B, T15 vs T25; C, T15-LN vs T15; D, T7 vs T7-d3. **Figure S10.** Schematic diagram of culture device.

## Data Availability

The authors promise the availability of supporting data.

## Abbreviations

EPA: Eicosapentaenoic acid
T7-d3: The algal cultures grown at temperature of 7 °C with 18 mM nitrogen treatment for 3 days
T7: The algal cultures grown at temperature of 7 °C with 18 mM nitrogen treatment for 12 days
T15: The algal cultures grown at temperature of 15 °C for 12 days with 18 mM nitrogen treatment
T15-LN: The algal cultures grown at temperature of 15 °C for 12 days with 3 mM nitrogen treatment
T25: The algal cultures grown at 25 °C with 18 mM nitrogen treatment for 12 days
T1: T7 vs T25
T2: T15 vs T25
T3: T15 vs T7
N: T15-LN vs T15
D: T7 vs T7-d3
AA: Arachidonic acid
DHA: Docosahexaenoic acid
MA: Myristic acid
PA: Palmitic acid
POA: Palmitoleic acid
LA: Linoleic acid
EEA: Eicosatrienoic acid
TFAs: Total fatty acids
TMT: Tandem mass tags
DEPs: Differentially expressed proteins
PSII: Photosystem II
PSI: Photosystem I
Rubisco: Ribulose-1,5-bisphosphate carboxylase/oxygenase
GK: Glucokinase
GPI: Glucose-6-phosphate isomerase
FBP: Fructose-1,6-bisphosphatase
PFK: 6-Phosphofructokinase
ALDO: Aldolase
GAPDH: Glyceraldehyde-3-phosphate dehydrogenase
PGK: Phosphoglycerate kinase
ENO: Enolase
PK: Pyruvate kinase
PC: Pyruvate carboxylase
PCK: Phosphoenolpyruvate carboxykinase
PDH: Pyruvate dehydrogenase
ACCase: Acetyl-coenzyme A carboxylase
MAT: Malonyl-ACP transferase
KAS: 3-Ketoacyl-ACP synthase
KAR: 3-Ketoacyl-ACP reductase
HAD: 3-Ketoacyl-ACP dehydrase
EAR: 3-Enyl-ACP reductase
G3P: Glycerate-3-phosphate
GPAT: Phosphatidic acid phosphatase;
DGAT: Diacylgycerol acyltransferase
PDAT: Phospholipid: diacylglcerol acyltransferase
FAT: Fatty-acyl-ACP thioesterases
KCS: 3-Ketoacyl-CoA synthase
KCR: Very-long-chain 3-oxoacyl-CoA reductase
HCD: Enoyl-CoA hydratase
ECR: Trans-2-enoyl-CoA reductase
PKC: Protein kinase C
PLC: Phospholipase C
IMP: Inositol monophosphatase
CaBP: Calcium binding protein
ROS: Reactive oxygen species
SOD: Superoxide dismutase
CAT: Catalase
GPX: Glutathione peroxidase
APX: Ascorbate peroxidase
POD: Peroxidase
CSPs: Cold shock proteins
TAG: Triacylglycerol

## References

[1] Schulze PS, Hulatt CJ, Morales-Sánchez D, Wijffels RH, Kiron V (2019). Fatty acids and proteins from marine cold adapted microalgae for biotechnology. Algal Res.

[2] Aratboni HA, Rafiei N, Garcia-Granados R, Alemzadeh A, Morones-Ramírez JR (2019). Biomass and lipid induction strategies in microalgae for biofuel production and other applications. Microb Cell Fact.

[3] Salama ES, Hwang JH, El-Dalatony MM, Kurade MB, Kabra AN, Abou-Shanab RA, Jeon BH (2018). Enhancement of microalgal growth and biocomponent-based transformations for improved biofuel recovery: a review. Bioresour Technol.

[4] Ermilova E (2020). Cold stress response: an overview in *Chlamydomonas*. Front Plant Sci.

[5] Leya T (2020). The CCCryo culture collection of cryophilic algae as a valuable bioresource for algal biodiversity and for novel, industrially marketable metabolites. Appl Phycol.

[6] Furhan J (2020). Adaptation, production, and biotechnological potential of cold-adapted proteases from psychrophiles and psychrotrophs: recent overview. J Genet Eng Biotechno.

[7] Cvetkovska M, Hüner NP, Smith DR (2017). Chilling out: the evolution and diversification of psychrophilic algae with a focus on *Chlamydomonadales*. Polar Biol.

[8] Mou S, Xu D, Ye N, Zhang X, Liang C, Liang Q, Zhen Z, Zhuang Z, Miao J (2012). Rapid estimation of lipid content in an Antarctic ice alga (*Chlamydomonas* sp.) using the lipophilic fluorescent dye BODIPY505/515. J Appl Phycol.

[9] Zou S, Huang Z, Wu X, Yu X (2022). Physiological and genetic regulation for high lipid accumulation by *Chlorella sorokiniana* strains from different environments of an arctic glacier, desert, and temperate lake under nitrogen deprivation conditions. Microbiol Spectr.

[10] Varshney P, Mikulic P, Vonshak A (2015). Extremophilic micro-algae and their potential contribution in biotechnology. Biores Technol.

[11] Guo F, Wang H, Wang J, Zhou W, Gao L, Chen L, Liu T (2014). Special biochemical responses to nitrogen deprivation of filamentous oleaginous microalgae *Tribonema* sp. Bioresour Technol.

[12] Wang F, Chen J, Zhang C, Gao B (2020). Resourceful treatment of cane sugar industry wastewater by Tribonema minus towards the production of valuable biomass. Biores Technol.

[13] Zhang Z, Qu C, Yao R, Nie Y, Xu C, Miao J, Zhong B (2019). The parallel molecular adaptations to the Antarctic cold environment in two psychrophilic green algae. Genome Biol Evol.

[14] Wang H, Zhang Y, Zhou W, Noppol L, Liu T (2018). Mechanism and enhancement of lipid accumulation in filamentous oleaginous microalgae *Tribonema minus* under heterotrophic condition. Biotechnol Biofuels.

[15] Tanaka T, Maeda Y, Veluchamy A, Tanaka M, Abida H, Maréchal E, Fujibuchi W (2015). Oil accumulation by the oleaginous diatom Fistulifera solaris as revealed by the genome and transcriptome. Plant Cell.

[16] Harwood JL, Guschina IA (2009). The versatility of algae and their lipid metabolism. Biochimie.

[17] Li J, Han D, Wang D, Ning K, Jia J, Wei L, Jing X, Huang S, Chen J, Li Y, Hu Q, Xu J (2014). Choreography of transcriptomes and lipidomes of *Nannochloropsis* reveals the mechanisms of oil synthesis in microalgae. Plant Cell.

[18] Chapman KD, Ohlrogge JB (2012). Compartmentation of triacylglycerol accumulation in plants. J Biol Chem.

[19] Li Y, Han D, Yoon K, Zhu S, Sommerfeld M, Hu Q, Richmond A, Hu Q (2013). Molecular and cellular mechanisms for lipid synthesis and accumulation in microalgae: biotechnological implications. Handbook of microalgal culture.

[20] An M, Mou S, Zhang X, Ye N, Zheng Z, Cao S, Xu D, Fan X, Wang Y, Miao J (2013). Temperature regulates fatty acid desaturases at a transcriptional level and modulates the fatty acid profile in the Antarctic microalga *Chlamydomonas* sp. ICE-L Bioresour Technol.

[21] Zheng SZ, Liu YL, Li B (2012). Phosphoinositide-specific phospholipase C9 is involved in the thermotolerance of *Arabidopsis*. Plant J.

[22] Halet G (2004). PKC signaling at fertilization in mammalian eggs. Biochem Biophys Acta.

[23] Li L, Peng H, Tan S, Zhou J, Chen L (2020). Effects of early cold stress on gene expression in *Chlamydomonas*
*reinhardtii*. Genomics.

[24] Nourbakhsh A, Collakova E, Gillaspy GE (2015). Characterization of the inositol monophosphatase gene family in Arabidopsis. Front Plant Sci.

[25] Barati B, Gan SY, Lim PE, Beardall J, Phang SM (2019). Green algal molecular responses to temperature stress. Acta Physiol Plant.

[26] Lyon BR, Mock T (2014). Polar microalgae: new approaches towards understanding adaptations to an extreme and changing environment. Biology.

[27] Ras M, Steyer JP, Bernard O (2013). Temperature effect on microalgae: a crucial factor for outdoor production. Rev Environ Sci Bio/Technol.

[28] Freire I, Cortina-Burgueño A, Grille P (2016). Nannochloropsis limnetica: a freshwater microalga for marine aquaculture. Aquaculture.

[29] Wei L, Huang X, Huang Z (2015). Temperature effects on lipid properties of microalgae *Tetraselmis* subcordiformis and *Nannochloropsis*
*oculata* as biofuel resources. Chin J Oceanol Limnol.

[30] Singh SP, Singh P (2015). Effect of temperature and light on the growth of algae species: a review. Renew Sustain Energy Rev.

[31] Reay DS, Nedwell DB, Priddle J, Ellis-Evans JC (1999). Temperature dependence of inorganic nitrogen uptake: reduced affinity for nitrate at suboptimal temperatures in both algae and bacteria. Appl Environ Microbiol.

[32] Lee N, Oh HS, Oh HM, Kim HS, Ahn CY (2019). Enhanced growth and lipid production in psychrotolerant *Acutodesmus* by controlling temperature-dependent nitrogen concentration. Biomass Bioenerg.

[33] Juneja A, Ceballos RM, Murthy GS (2013). Effects of environmental factors and nutrient availability on the biochemical composition of algae for biofuels production: a review. Energies.

[34] Fan J, Cui Y, Wan M, Wang W, Li Y (2014). Lipid accumulation and biosynthesis genes response of the oleaginous *Chlorella pyrenoidosa* under three nutrition stressors. Biotechnol Biofuels.

[35] Bartley ML, Boeing WJ, Corcoran AA, Holguin FO, Schaub T (2013). Effects of salinity on growth and lipid accumulation of biofuel microalga *Nannochloropsis salina* and invading organisms. Biomass Bioenerg.

[36] Elsayed KNM, Kolesnikova TA, Noke A, Klock G (2017). Imaging the accumulated intracellular microalgal lipids as a response to temperature stress. 3 Biotech.

[37] Suzuki H, Hulatt CJ, Wijffels RH, Kiron V (2019). Growth and LC-PUFA production of the cold-adapted microalga *Koliella antarctica* in photobioreactors. J Appl Phycol.

[38] Koh HG, Kang NK, Kim EK, Suh WI, Park WK, Lee B, Chang YK (2019). Isolation and characterization of novel *Chlorella* species with cold resistance and high lipid accumulation for biodiesel production. J Microbiol Biotechnol.

[39] Hulatt CJ, Berecz O, Egeland ES, Wijffels RH, Kiron V (2017). Polar snow algae as a valuable source of lipids. Biores Technol.

[40] Valledor L, Furuhashi T, Hanak AM, Weckwerth W (2013). Systemic cold stress adaptation of *Chlamydomonas reinhardtii*. Mol Cell Proteomics.

[41] Xue WB, Liu F, Sun Z, Zhou ZG (2016). A delta-9 fatty acid desaturase gene in the microalga *Myrmecia incisa* reisigl: cloning and functional analysis. Int J Mol Sci.

[42] Liu J, Sun Z, Zhong Y, Huang J, Hu Q, Chen F (2012). Stearoyl-acyl carrier protein desaturase gene from the oleaginous microalga Chlorella zofingiensis: cloning, characterization and transcriptional analysis. Planta.

[43] Collins T, Margesin R (2019). Psychrophilic lifestyles: mechanisms of adaptation and biotechnological tools. Applied Microbiol Biotechnol.

[44] Russell NJ, Margesin R, Schinner F, Marx J-C, Gerday C (2008). Membrane components and cold sensing. Psychrophiles: from biodiversity to biotechnology.

[45] Miguez F, Schiefelbein U, Karsten U, Garcia-Plazaola JI, Gustavs L (2017). Unraveling the photoprotectiv response of lichenized and free-living green algae (*Trebouxiophyceae*, Chlorophyta) to photochilling stress. Front Plant Sci.

[46] Chong GL, Chu WL, Othman RY, Phang SM (2011). Differential gene expression of an Antarctic *Chlorella* in response to temperature stress. Polar Biol.

[47] Mock T, Hoch N (2005). Long-term temperature acclimation of photosynthesis in steady-state cultures of the polar diatom *Fragilariopsis cylindrus*. Photosyn Res.

[48] Deng Y, Zhan Z, Tang X, Ding L, Duan D (2014). Molecular cloning and expression analysis of RbcL cDNA from the bloom-forming green alga *Chaetomorpha valida* (Cladophorales, Chlorophyta). J Appl Phycol.

[49] Clare DA, Rabinowitch HD, Fridovich I (1984). Superoxide dismutase and chilling injury in *Chlorella ellipsoidea*. Arch Biochem Biophys.

[50] Schriek R (2000). Effects of light and temperature on the enzymatic antioxidative defense systems in the Antarctic ice diatom *Entomoneis kufferathii* Manguin. Rep Polar Res.

[51] Gupta SK, Kataki S, Chatterjee S, Prasad RK, Datta S, Vairale MG, Gupta DK (2020). Cold adaptation in bacteria with special focus on cellulase production and its potential application. J Clean Prod.

[52] Ricciardelli A, Casillo A, Vergara A, Balasco N, Corsaro MM, Tutino ML, Parrilli E (2019). Environmental conditions shape the biofilm of the antarctic bacterium *Pseudoalteromonas*
*haloplanktis* TAC125. Microbiol Res.

[53] Schmeing TM, Voorhees RM, Kelley AC (2009). The crystal structure of the ribosome bound to EF-Tu and aminoacyl-tRNA. Science.

[54] Casanueva A, Tuffin M, Cary C, Cowan DA (2010). Molecular adaptations to psychrophily: the impact of ‘omic’ technologies. Trends Microbiol.

[55] Zhang Y, Burkhardt DH, Rouskin S, Li GW, Weissman JS, Gross CA (2018). A stress response that monitors and regulates mRNA structure is central to cold shock adaptation. Mol Cell.

[56] Kawamoto J, Kurihara T, Esaki N, Margesin R (2017). Proteomic insights of psychrophiles. Psychrophiles: from biodiversity to biotechnology.

[57] Gao B, Wang F, Huang L, Liu H, Zhong Y, Zhang C (2021). Biomass, lipid accumulation kinetics, and the transcriptome of heterotrophic oleaginous microalga *Tetradesmus bernardii* under different carbon and nitrogen sources. Biotechnol Biofuels.

[58] Jorrin-Novo JV, Jorrin-Novo JV, Komatsu S, Weckwerth W, Wienkoop S (2014). Plant proteomics methods and protocols. Plant proteomics.

[59] Wiśniewski JR, Zougman A, Nagaraj N (2009). Universal sample preparation method for proteome analysis. Nat Methods.

[60] Perez-Riverol Y, Bai J, Bandla C, Hewapathirana S, García-Seisdedos D, Kamatchinathan S, Kundu D, Prakash A, Frericks-Zipper A, Eisenacher M, Walzer M, Wang S, Brazma A, Vizcaíno JA (2022). The PRIDE database resources in 2022: a hub for mass spectrometry-based proteomics evidences. Nucleic Acids Res.

