# Supplementary material for: The growth, lipid accumulation and adaptation mechanism in response to variation of temperature and nitrogen supply in psychrotrophic filamentous microalga Xanthonema hormidioides (Xanthophyceae)
Source: Biotechnol Biofuels Bioprod. 2023 Jan 19;16:12. doi: 10.1186/s13068-022-02249-0 (PMC9854199; doi:10.1186/s13068-022-02249-0)
Supplement: Supplementary file 1 — Additional file 1: Figure S1. Cellular morphology of Xanthonema hormidioides; The scale bars indicate 10 μm. Figure S2. The effect of three nitrogen concentrations on the growth, consumption of medium nitrogen concentration and lipid content of X. hormidioides under different temperatures; A, C, E, 5 °C; B, D, F, 30 °C. Figure S3. The effect of different temperature on the consumption of medium phosphorus concentration; A, 5 °C; B, 7 °C; C, 10 °C; D, 15 °C; E, 20 °C; F; 25 °C; G, 27 °C; H, 30 °C. Figure S4. Fatty acid profiles of X. hormidioides in terms of dry weight under different nitrogen concentrations and temperatures (the three columns of each time point show different nitrogen concentration treatment, from left to right as 3, 9 and 18 mM nitrogen treatment, respectively; A, 7 °C; B, 10 °C; C, 15 °C; D, 20 °C; E, 25 °C; F; 27 °C). Figure S5. Fatty acid profiles of X. hormidioides in terms of total fatty acids under different nitrogen concentrations and temperatures. Figure S6. GO analysis of differentially expressed proteins of X. hormidioides. Figure S7. KEGG analysis of differentially expressed proteins of X. hormidioides. Figure S8. The expression of ribosomal proteins among different comparison groups; Red, up-regulated; Green, down-regulated; A, T7 vs T25; B, T15 vs T25; C, T15-LN vs T15; D, T7 vs T7-d3. Figure S9. The expression of photosynthesis related proteins among different comparison groups; Red, up-regulated; Green, down-regulated; A, T7 vs T25; B, T15 vs T25; C, T15-LN vs T15; D, T7 vs T7-d3. Figure S10. Schematic diagram of culture device. [file 13068_2022_2249_MOESM1_ESM.docx]

**
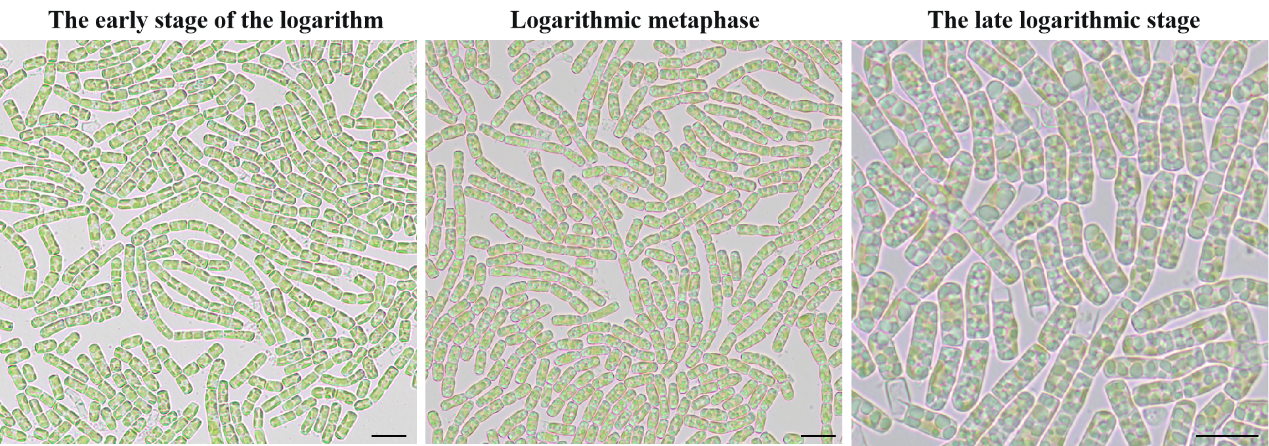
**

Fig. S1 Cellular morphology of *Xanthonema hormidioides*; The scale bars indicate 10 μm


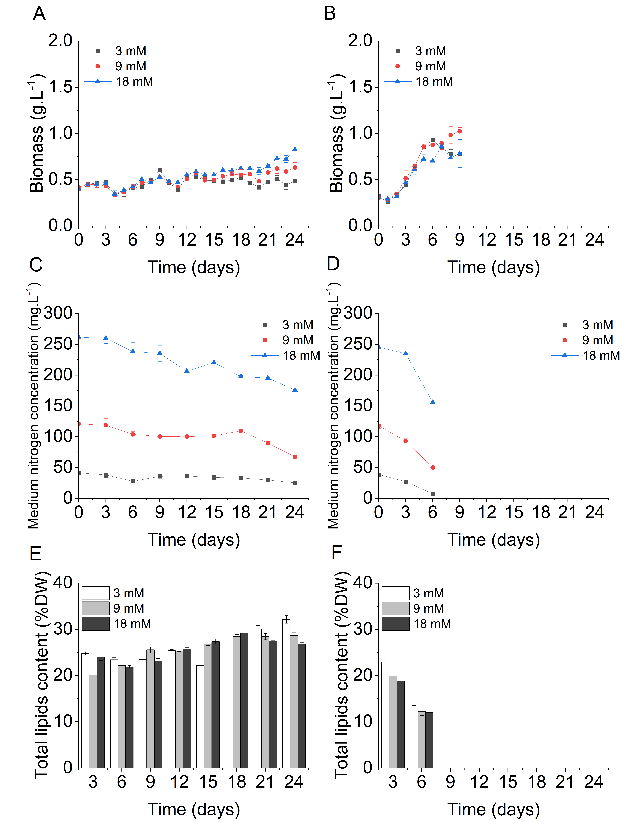


Fig. S2 The effect of three nitrogen concentrations on the growth, consumption of medium nitrogen concentration and lipid content of *X. hormidioides* under different temperatures; A, C, E, 5℃; B, D, F, 30℃


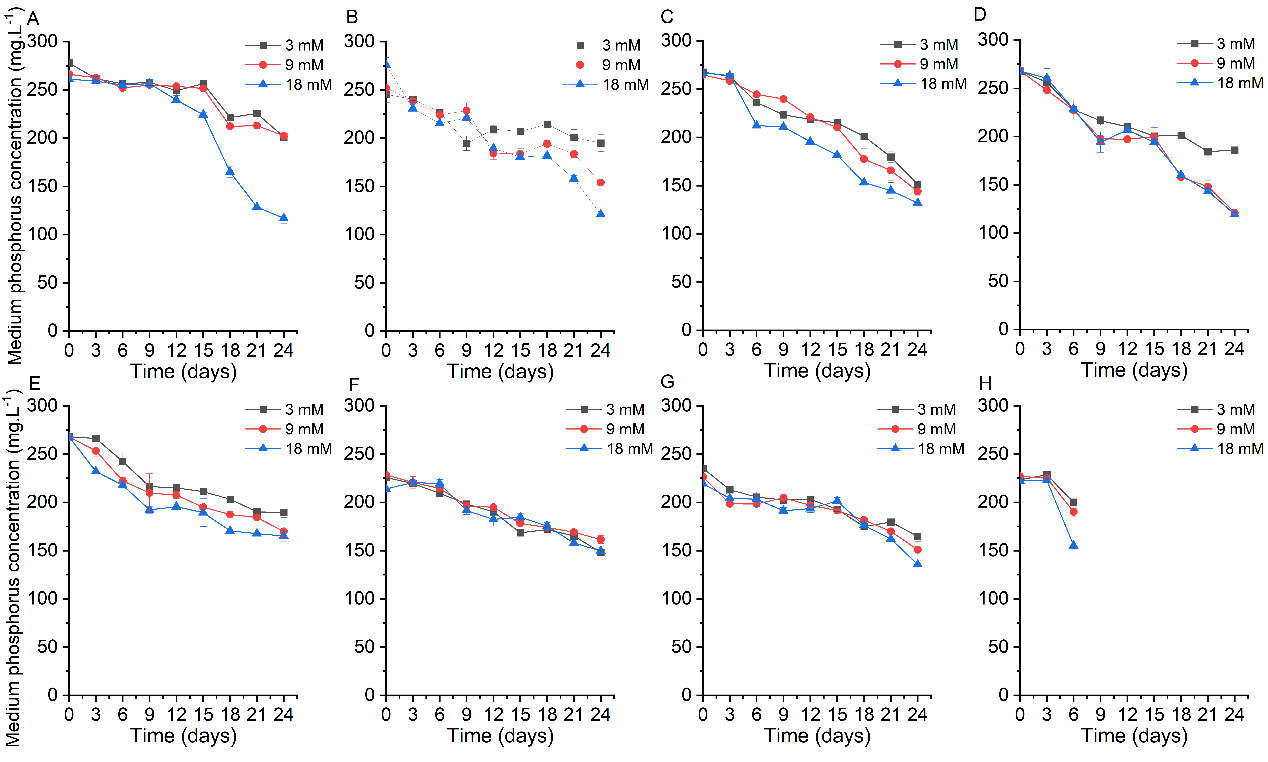


Fig. S3 The effect of different temperature on the consumption of medium [phosphorus](javascript:;) concentration; A, 5℃; B, 7℃; C, 10℃; D, 15℃; E, 20℃; F; 25℃; G, 27℃; H, 30℃


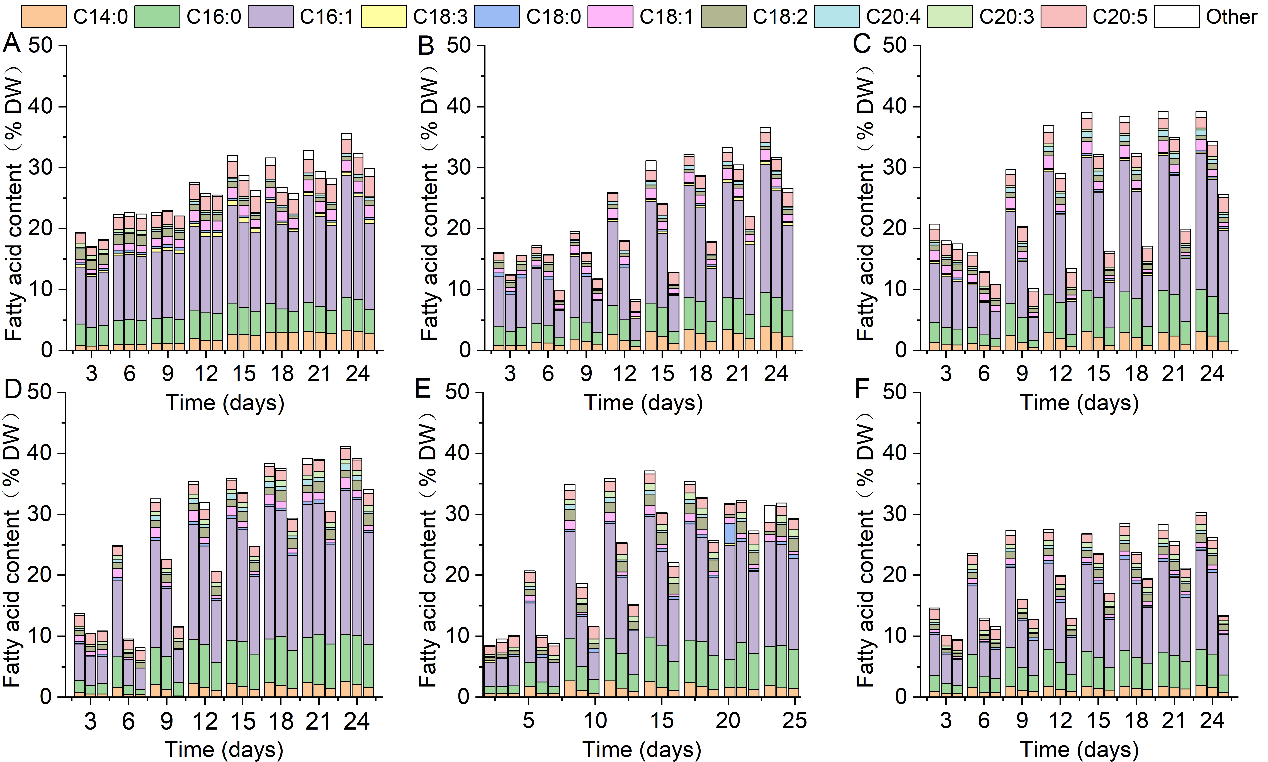


Fig. S4 Fatty acid profiles of *X. hormidioides* in terms of dry weight under different nitrogen concentrations and temperatures (the three columns of each time point show different nitrogen concentration treatment, from left to right as 3, 9 and 18 mM nitrogen treatment, respectively; A, 7℃; B, 10℃; C, 15℃; D, 20℃; E, 25℃; F; 27℃)


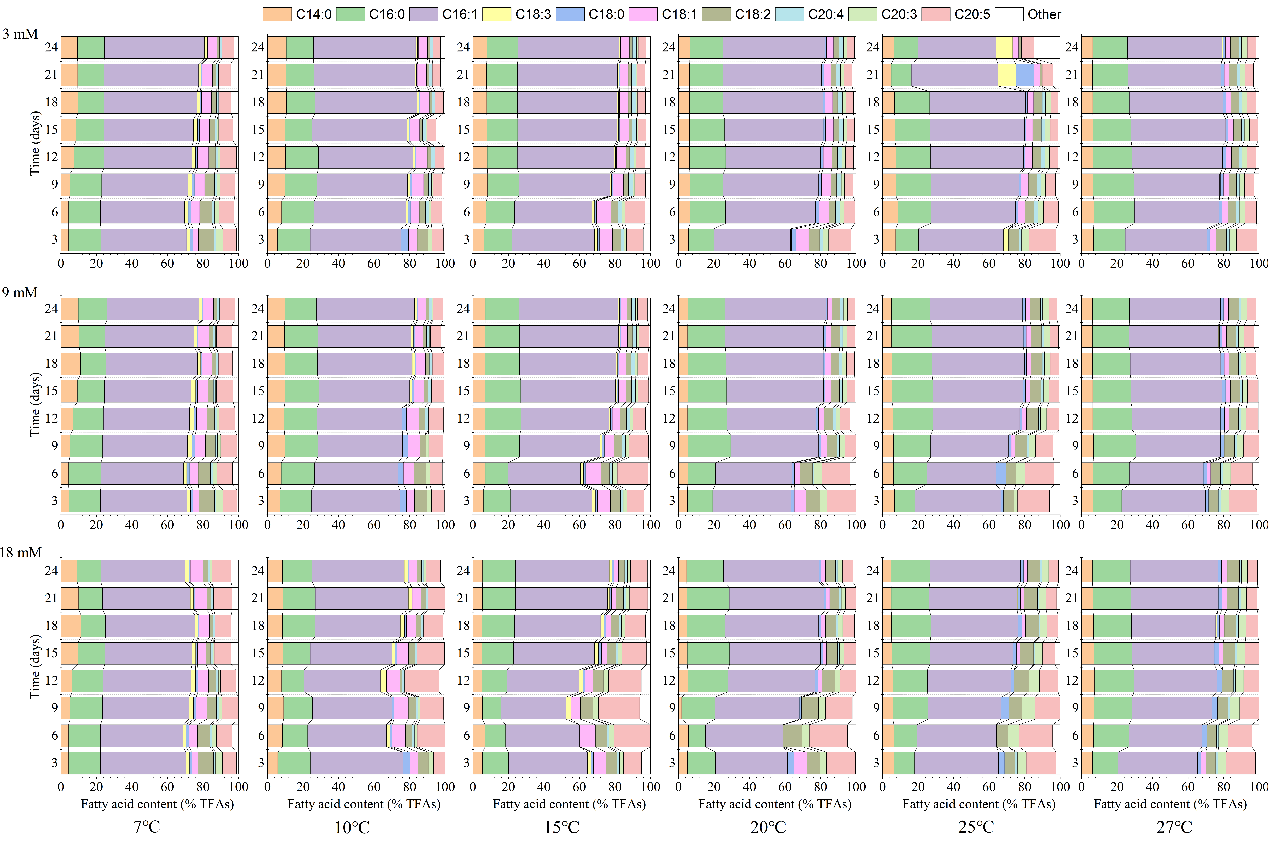


Fig. S5 Fatty acid profiles of *X. hormidioides* in terms of total fatty acids under different nitrogen concentrations and temperatures


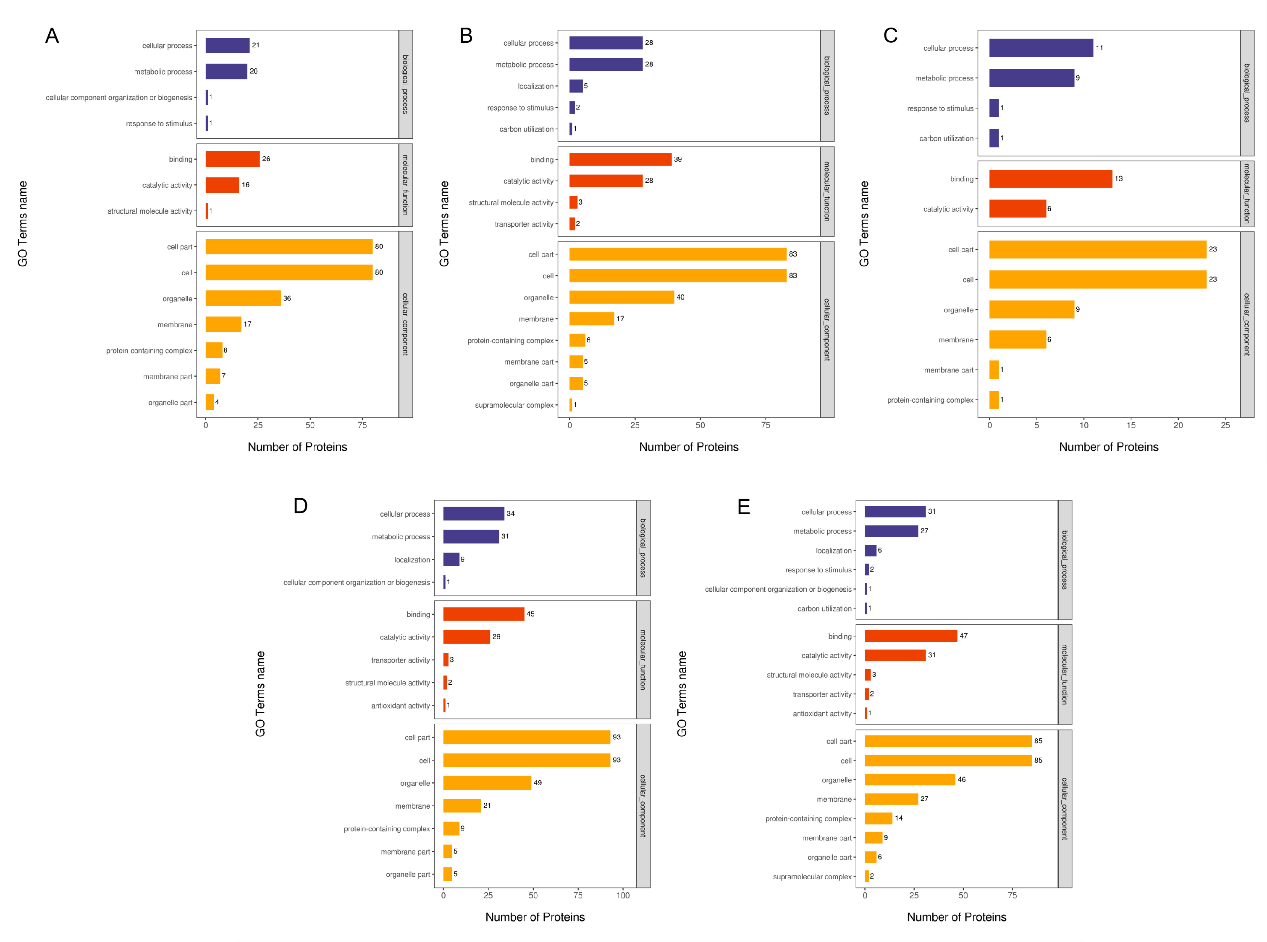


Fig. S6 GO analysis of differentially expressed proteins of *X. hormidioides*

*
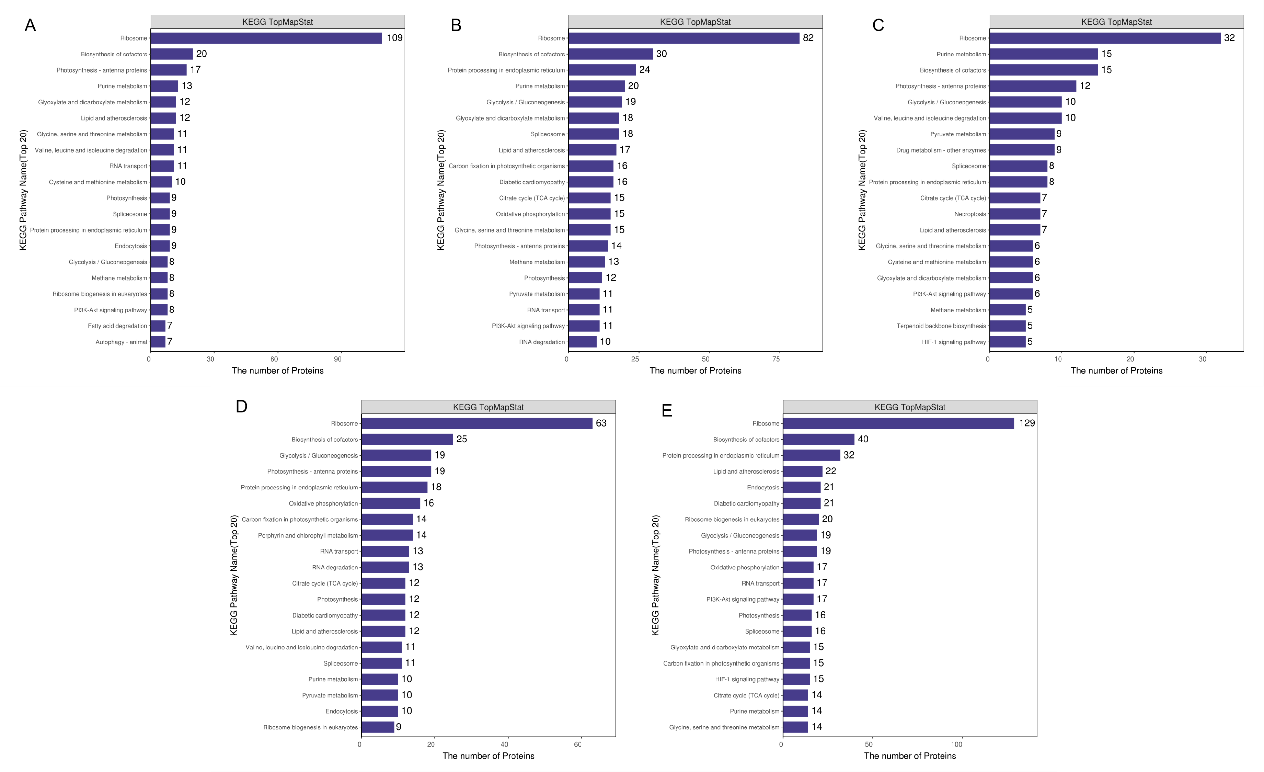
*

Fig. S7 KEGG analysis of differentially expressed proteins of *X. hormidioides*


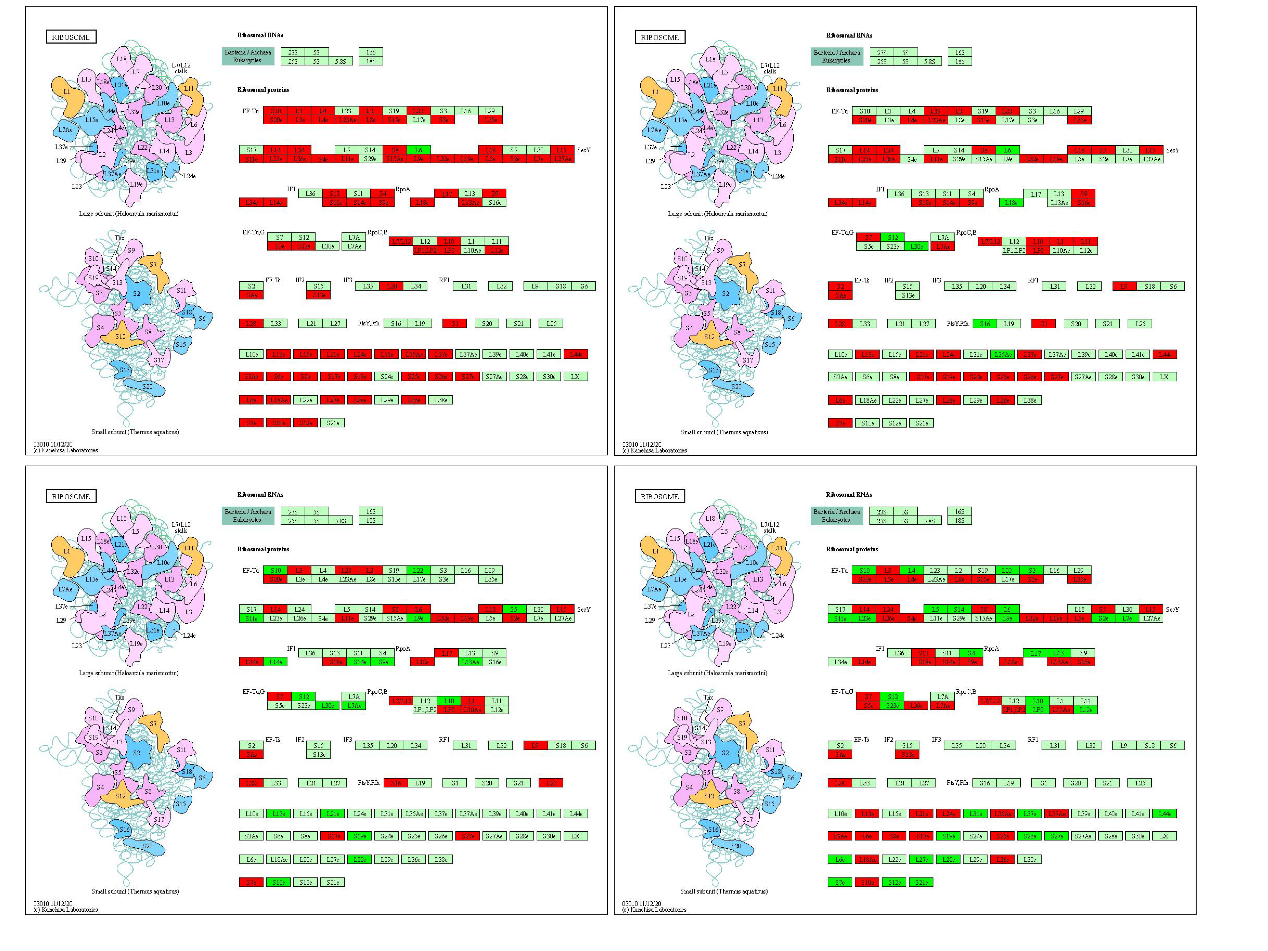


Fig. S8 The expression of ribosomal proteins among different comparison groups; Red, up-regulated; Green, down-regulated; A, T7 vs T25; B, T15 vs T25; C, T15-LN vs T15; D, T7 vs T7-d3


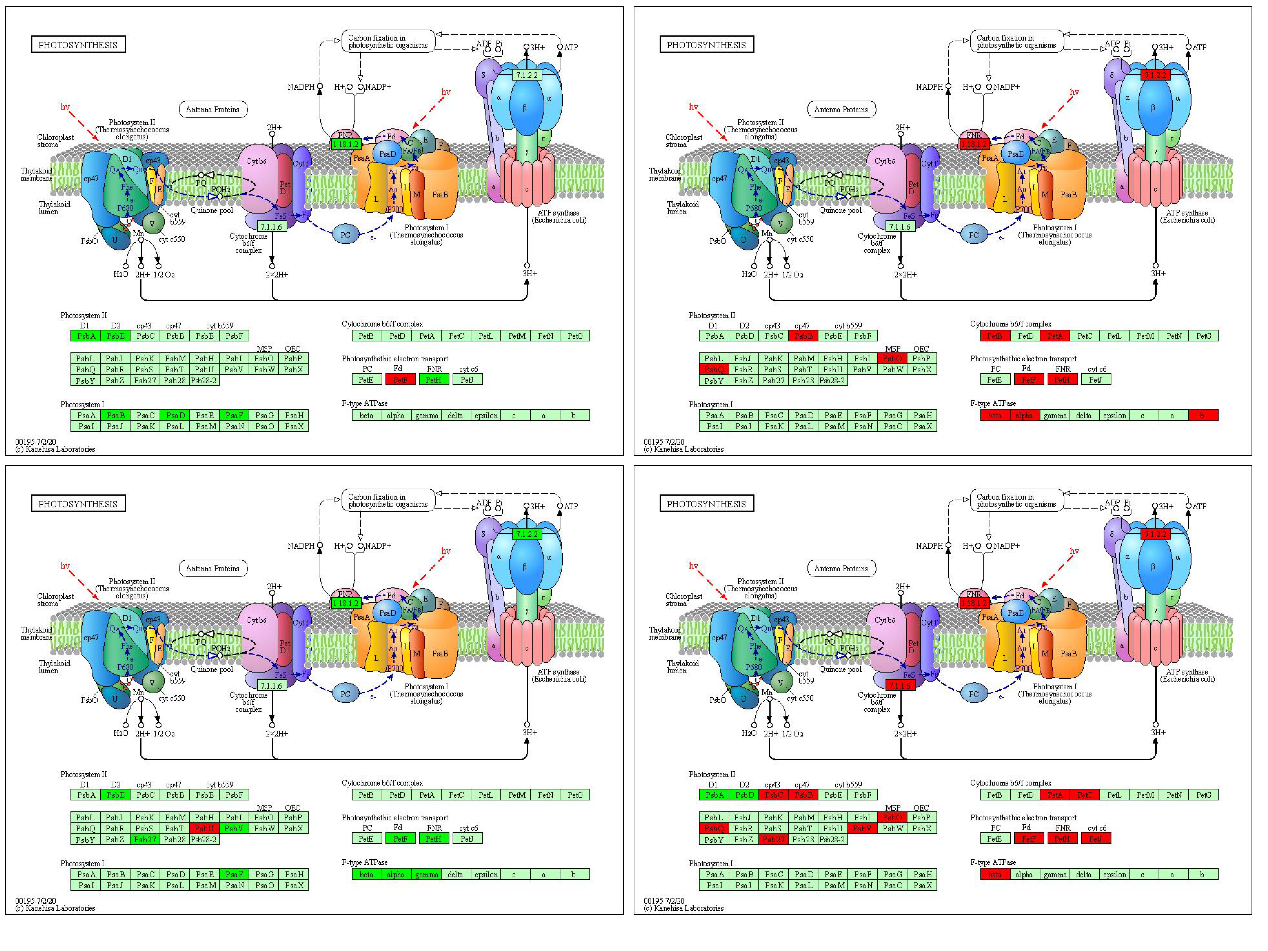


Fig. S9 The expression of photosynthesis related proteins among different comparison groups; Red, up-regulated; Green, down-regulated; A, T7 vs T25; B, T15 vs T25; C, T15-LN vs T15; D, T7 vs T7-d3


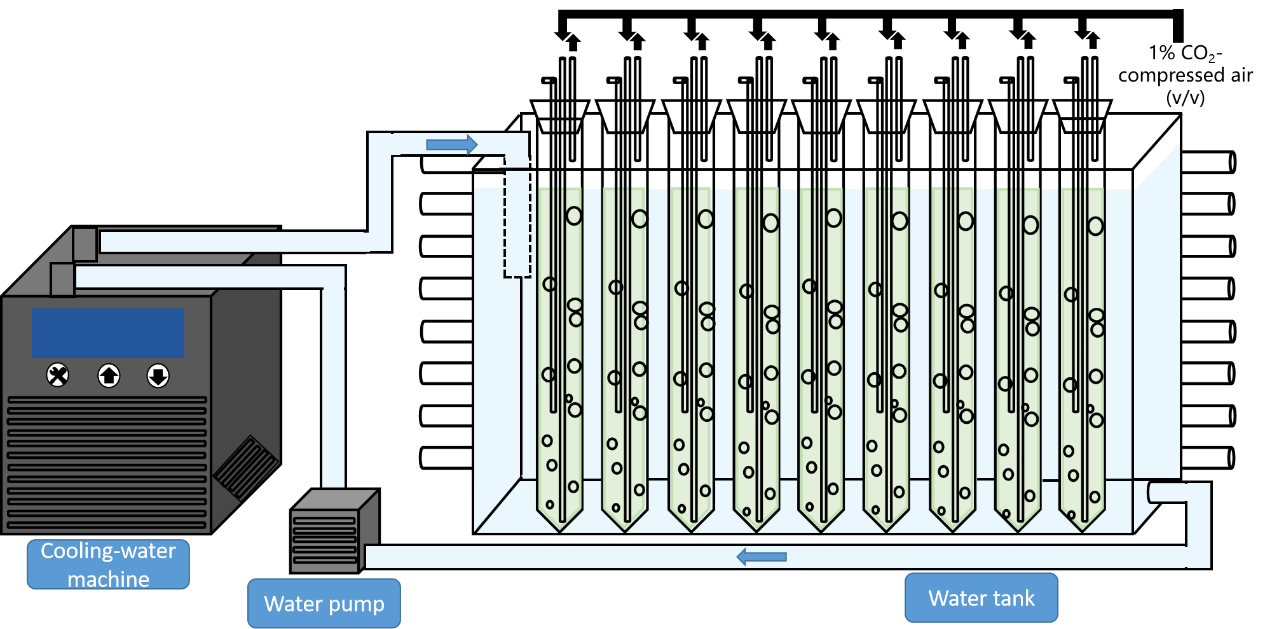


Fig. S10 Schematic diagram of culture device
